# A Modular Cytokine Analysis Method Reveals Novel Associations With Clinical Phenotypes and Identifies Sets of Co-signaling Cytokines Across Influenza Natural Infection Cohorts and Healthy Controls

**DOI:** 10.3389/fimmu.2019.01338

**Published:** 2019-06-18

**Authors:** Liel Cohen, Andrew Fiore-Gartland, Adrienne G. Randolph, Angela Panoskaltsis-Mortari, Sook-San Wong, Jacqui Ralston, Timothy Wood, Ruth Seeds, Q. Sue Huang, Richard J. Webby, Paul G. Thomas, Tomer Hertz

**Affiliations:** ^1^Department of Industrial Engineering and Management, Ben-Gurion University of the Negev, Be'er-Sheva, Israel; ^2^National Institute for Biotechnology in the Negev, Ben-Gurion University of the Negev, Be'er-Sheva, Israel; ^3^Vaccine and Infectious Disease Division, Fred Hutchinson Cancer Research Center, Seattle, WA, United States; ^4^Department of Anesthesiology, Critical Care and Pain Medicine, Boston Children's Hospital, Boston, MA, United States; ^5^Departments of Anaesthesia and Pediatrics, Harvard Medical School, Boston, MA, United States; ^6^Department of Pediatrics, Bone Marrow Transplantation, Pulmonary and Critical Care Medicine, University of Minnesota, Minneapolis, MN, United States; ^7^State Key Laboratory of Respiratory Diseases, Guangzhou Medical University, Guangzhou, China; ^8^Institute for Environmental Science and Research, National Centre for Biosecurity and Infectious Disease, Upper Hutt, New Zealand; ^9^Department of Infectious Diseases, St. Jude Children's Research Hospital, Memphis, TN, United States; ^10^Department of Immunology, St. Jude Children's Research Hospital, Memphis, TN, United States; ^11^Department of Microbiology, Immunology and Genetics, Ben-Gurion University of the Negev, Be'er-Sheva, Israel

**Keywords:** innate immunology, cytokines, chemokines, influenza, biomarker

## Abstract

Cytokines and chemokines are key signaling molecules of the immune system. Recent technological advances enable measurement of multiplexed cytokine profiles in biological samples. These profiles can then be used to identify potential biomarkers of a variety of clinical phenotypes. However, testing for such associations for each cytokine separately ignores the highly context-dependent covariation in cytokine secretion and decreases statistical power to detect associations due to multiple hypothesis testing. Here we present CytoMod—a novel data-driven approach for analysis of cytokine profiles that uses unsupervised clustering and regression to identify putative functional modules of co-signaling cytokines. Each module represents a biosignature of co-signaling cytokines. We applied this approach to three independent clinical cohorts of subjects naturally infected with influenza in which cytokine profiles and clinical phenotypes were collected. We found that in two out of three cohorts, cytokine modules were significantly associated with clinical phenotypes, and in many cases these associations were stronger than the associations of the individual cytokines within them. By comparing cytokine modules across datasets, we identified cytokine “cores”—specific subsets of co-expressed cytokines that clustered together across the three cohorts. Cytokine cores were also associated with clinical phenotypes. Interestingly, most of these cores were also co-expressed in a cohort of healthy controls, suggesting that in part, patterns of cytokine co-signaling may be generalizable. CytoMod can be readily applied to any cytokine profile dataset regardless of measurement technology, increases the statistical power to detect associations with clinical phenotypes and may help shed light on the complex co-signaling networks of cytokines in both health and infection.

## 1. Introduction

Cytokines and chemokines are key signaling molecules of the immune system, mediating a complex network of interacting cells that govern the immune response (1, 2). These small proteins secreted by a broad range of cells, regulate host responses to infection, trauma and sepsis and are involved in inflammatory and autoimmune diseases. The role of cytokines in disease as well as the associations between cytokine production levels and the occurrence of diseases and their phenotypes has been extensively studied (3), and many studies have shown that cytokine signaling is context-dependant (4). Cytokine expression and dysregulation have been linked with a variety of diseases such as diabetes (5, 6), Alzheimer's (7), cancer (8–11), heart disease (12, 13), and various viral infections including influenza, EBV, RSV, HIV and dengue (14–18).

Influenza is a respiratory virus that accounts for significant rates of hospitalizations and deaths, especially among very young or old individuals (19). Due to the variety of influenza subtypes and their rapid evolution, influenza causes annual epidemics and occasional catastrophic pandemics (20, 21). Influenza infection in humans can result in asymptomatic to serious illness with symptoms such as fever, myalgia, headache and upper and lower respiratory symptoms. The respiratory tract infection can progress to various acute conditions, e.g., pneumonia and acute respiratory distress syndrome (ARDS) or a “cytokine storm” causing widespread tissue damage (22, 23). In some cases, complications are caused by a secondary bacterial infection such as *Staphylococcus aureus*.

Cytokine expression in response to influenza infection has been studied using human blood and nasal samples, immune cell cultures and animal models (23, 24). Numerous studies have reported associations of individual cytokines with various influenza phenotypes and outcomes such as hospitalization and death. Each study tested a specific subset of cytokines. From these studies, several prominent cytokines have been repeatedly found to be associated with illness and symptoms including IL-6, TNF-α, IL-10, IL-8, IP-10, IFN-γ, and MCP-1 (23–32). Differences in cytokine expression levels were found between subjects infected with different Influenza strains, as well as different severity and symptoms. For example, the H5N1 strains were found to induce high serum levels of IP-10 and monokine induced by interferon-γ (MIG) (25, 33) and also higher levels of TNF-α and IFN-β compared to H3N2 or H1N1 strains (29). Another study reported hyperactivation of IL-6, IL-8, and MCP-1 in blood of subjects infected with pandemic H1N1 that developed pneumonia and in complicated seasonal influenza, but not in milder pandemic H1N1 infections (28). A significant correlation has been reported between disease severity and the levels of IL-6, IL-10, and IL-15 (32), and in contrast, IL-17 was lower in more severe patients (28, 32).

Despite our partial understanding of cytokine biology there are a variety of therapeutic treatments that target specific cytokines, which are in wide clinical use to treat autoimmune diseases and cancer. There are a variety of licensed monoclonal antibody (Ab) treatments that target cytokines or their receptors. Examples include: anti TNF-α Abs (34, 35), an anti IL-6 receptor Ab (35, 36), anti IL-1 Abs (35), anti IL-10 Abs (37), anti IL-23 Abs (38), and anti Herceptin Abs (39). Most notably, Humira-an anti TNF-α Ab is widely used to treat a variety of autoimmune diseases and was the best selling drug in 2017 (40).

Since cytokines and chemokines (hereafter referred to as cytokines) reflect the local or systemic immune state, they have the potential to serve as indicators of various clinical conditions. Various studies suggested the use of measurements of circulating cytokines as biomarkers in order to aid clinicians in patient prognosis and care (41–46). Furthermore, as the understanding of cytokine biology improves, new treatment strategies emerge to leverage this knowledge (47). Several methodologies have been developed for quantification of secreted cytokines in body fluid samples, including immunoassays such as ELISA and bead-based multiplex immunoassays (48), allowing the collection and analysis of cytokine “profiles”: a broad and unbiased assessment of cytokine levels that typically includes 10–50 cytokines of interest.

While numerous studies have reported associations between cytokine levels and various clinical phenotypes, the analysis of cytokine profiles is often statistically underpowered to detect such associations, due to the large number of cytokines and the requirement for multiplicity adjustment. Furthermore, the relatively high-cost of cytokine profiles limits the sizes of cohorts for which they are measured. A typical cytokine profile dataset can have measurements obtained from tens to hundreds of subjects. These opposing trends make it increasingly important to develop new computational tools for analyzing cytokine profiles that are statistically efficient and provide interpretable results.

One possible solution for preserving statistical power, is to select a small subset of cytokines for a primary analysis with phenotypes, with a secondary/exploratory analysis that includes all remaining cytokines. For example, in previous work on cytokine profiles following influenza natural infection we pre-selected a subset of 11 cytokines for the primary analyses based on published studies (49). Multiplicity adjustment was performed across the 11 cytokines pre-selected in our analysis plan. While this approach identified several significant associations with phenotypes, it failed to detect other significant associations of cytokines that were not selected in the primary set as we demonstrate below. Furthermore, it required pre-existing knowledge for selecting the primary set of cytokines, limiting the ability to discover novel associations.

Another important property of cytokine signaling is its inherent redundancy (2, 50). Many of the same cytokines may be simultaneously secreted by different immune cells, and activation or attenuation of an immune pathway can often be mediated by multiple cytokines. Therefore, cytokine profiles typically exhibit high levels of pairwise correlations among cytokines, across subjects, as previously demonstrated (49, 51). These complexities pose particular challenges in the interpretation and analysis of cytokine data by practitioners.

**Figure 1:**
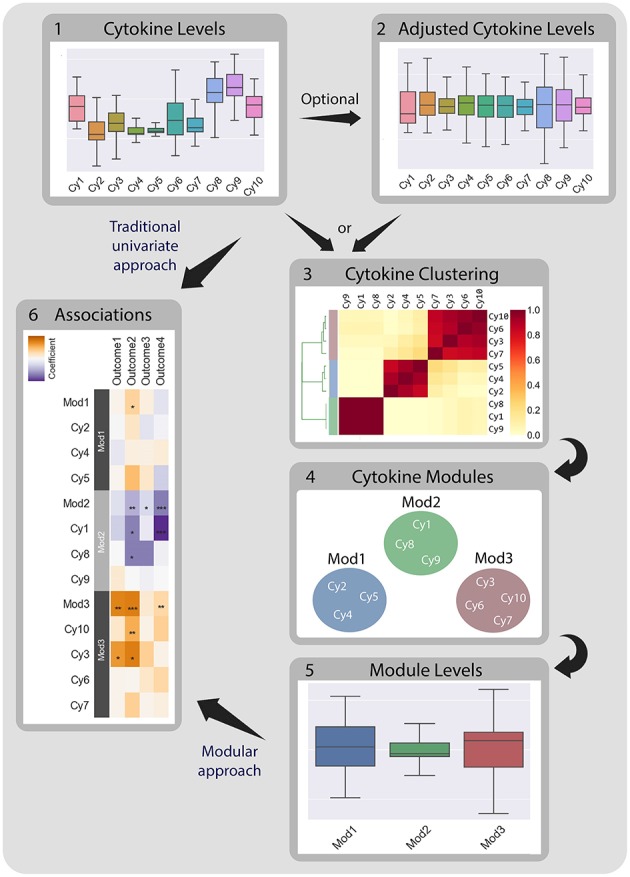
CytoMod—a modular data driven approach to identify cytokine modules and assess their associations with clinical phenotypes. Traditionally, associations between cytokine data (1) and clinical phenotypes (5) are tested directly using univariate models. CytoMod independently uses absolute cytokine profiles (1) or adjusted cytokine profiles (2) to generate cytokine modules (3)-sets of co-signaling cytokines within a given cohort. Modules are generated using unsupervised hierarchical clustering. Associations are then tested between module levels (4) and clinical phenotypes (5). By significantly reducing the number of associations tested CytoMod increases the statistical power to detect associations. By comparing modules across datasets, CytoMod can also identify “cores” of cytokines that consistently co-signal together.

Motivated by previous work, and the growing abundance of cytokine profile datasets, we developed CytoMod: a novel method for the analysis of cytokine profiles based on identifying cytokine modules. The modular-based approach is partly inspired by similar approaches used for analyzing gene expression data (52–57). Our proposed method aims to increase statistical power to detect associations of cytokines with clinical phenotypes by grouping cytokines into putative functional modules, using a data-driven clustering approach. Cytokines are grouped based on their pairwise correlations using hierarchical clustering. Modules are formed over absolute and adjusted cytokine levels. Associations are then assessed between cytokine modules and phenotypes as opposed to individual cytokines (Figure 1). An earlier version of this method was used to analyze cytokine profiles of influenza infected children that were admitted to the intensive care units (51). Here we extended this method to allow fully automated identification of modules and applied it to three independent clinical cohorts of natural influenza infection in which cytokine profiles were obtained and clinical phenotypes were collected. We found that in two of these cohorts, cytokine modules were significantly associated with clinical phenotypes, and in many cases these associations were stronger than the associations of the individual cytokines within each of the modules. Applying our method to these three independent cytokine profile cohorts we identified specific subsets of cytokines (cytokine “cores”) that clustered together across the three cohorts, and which were also associated with clinical phenotypes. These cytokine cores identify subsets of cytokines that are co-expressed during influenza infection, and most were also observed in healthy individuals. Our method can be readily applied to any cytokine profile dataset, and is publicly available for use using Python code or an interactive Jupyter Notebook.

## 2. Materials and Methods

### 2.1. Data

We analyzed cytokine profiles of 611 subjects collected from three independent studies (49, 51, 58) of subjects naturally infected with influenza virus as well as healthy controls: (1) PICFLU-a prospective multi-center study of children admitted to intensive care units with severe influenza infection (51); (2) FLU09-a prospective study of children admitted to the emergency room with influenza like-illness and their household members (49); and (3) The Southern Hemisphere Influenza and Vaccine Effectiveness Research and Surveillance (SHIVERS)—a prospective study of influenza infected subjects collected in New Zealand (58). Influenza positive cohorts included 221, 161, and 87 subjects, respectively, which were all tested and found positive for influenza (by DFA, PCR, RT-PCR, or culture). The FLU09 study also included 142 healthy control subjects.

**PICFLU** - The PICFLU study was a prospective multi-center study of severe influenza infections in children aged 0.06–18.19 years (median 6.97) (51). Blood samples were collected from a total of 221 children diagnosed with influenza critical illness that arrived at intensive care units (ICUs) at 35 hospitals between December 2008 and May 2015. An endotracheal sample was collected from all subjects that were intubated. Samples were provided at enrollment (mostly within 24 h of intensive care unit admission). Almost half of the enrolled subjects received vasoactive agents for septic shock and a similar fraction met criteria for Acute Respiratory Distress Syndrome (ARDS) with the majority having the severe form. Most subjects (*n* = 175, 79.2%) were influenza type A positive, while the remaining cases (*n* = 46, 20.8%) were influenza type B positive. Eighty-one subjects (36.6%) had a bacterial coinfection, predominantly with *Staphylococcus aureus, Streptococcus pneumoniae* and *Streptococcus pyogenes*. Additional information regarding the design, sampling, and subjects in PICFLU cohort can be found in Table 1.

**Table 1:** Characteristics and clinical information of patients from the analyzed cohorts.

**Cohort**	**PICFLU**	**FLU09 sick**	**FLU09 healthy**	**SHIVERS**
Sample size (n =)	221	166	142	87
Age	0.06–18.19	0.05–69.53	0.15–65.19	5–88
(median)	(6.97)	(6.29)	(24.77)	(48)
Gender				
Male	126	79	35	36
Female	95	87	107	51
Hospitalized	221	36	0	60
Intensive Care Unit	221	4	0	4
Sample type				
Blood	215	96	119	87
Airway	93	165	141	
Clinical phenotypes & outcomes				
Death	12	1	0	0
ECMO	9	–	–	–
Pneumonia-ARDS	100	–	–	–
Shock	103	–	–	–
Bacterial Coinfection	81	–	–	–
SARI	–	–	–	60
Influenza subtype / strain				
A (strain unknown)	29	6	0	16
A H1	92	68	0	0
A H3	53	62	0	45
B	46	27	0	26
Unknown	1	3	0	0

− means the outcome was not tracked in the study.

**FLU09** - The FLU09 study was a prospective study of children and their household members. It included samples of blood plasma and nasal swab/lavage from influenza infected subjects as well as their asymptomatic Influenza-positive household contacts. Three hundred and three subjects aged 0.05–69.53 years (median 17.23) were enrolled during 2009–2014 and included 142 healthy household members. A preliminary analysis of cytokine profiles (49) included only subjects from 2009 to 2011. Most samples were provided at enrollment and only few were taken within the first week. The cohort included 36 (22.4%) individuals who were hospitalized, four of them (2.5%) were admitted to the ICU. 5 (3.1%) suffered from febrile acute respiratory disease and another single subject (0.006%) had ARDS and died. Three subjects (1.8%) suffered from a bacterial coinfection. Study subjects were asked to rank their symptom severity daily according to a visual analog scale (VAS) until study completion. The symptoms considered were upper respiratory tract (URT) symptoms (sore throat, stuffy/runny nose, sinus fullness/facial pain); lower respiratory tract (LRT) symptoms (cough, shortness of breath, wheezing); systemic symptoms (feverishness, fatigue or malaise, headache, body aches or myalgia, chills, lethargy); gastrointestinal symptoms (nausea, vomiting, diarrhea). The FLU09 study also included 142 healthy control subjects for which cytokine profiles were also measured. These data were analyzed separately in section 3.5. Additional information regarding the design, sampling and subjects in FLU09 cohort can be found in Table 1.

**SHIVERS** - The Southern Hemisphere Influenza and Vaccine Effectiveness Research and Surveillance (SHIVERS) study included 87 Influenza infected subjects recruited from 16 sentinel general practices and 4 hospitals (58). Subjects were enrolled between 2013 and 2015 (aged 12–78 years, median 44.5). Sixty (68.9%) subjects recruited from hospitals demonstrated symptoms of Severe Acute Respiratory Illness (SARI), defined as “an acute respiratory illness with a history of fever or measured fever of ≥ 38°C, and cough, and onset within the past 10 days, and requiring inpatient hospitalization” (59), while the rest suffered from a milder form of Acute Respiratory Illness (ARI), i.e., do not require hospitalization (59). The majority of the SARI cases were relatively mild. Four subjects (4.59%) were admitted to the ICU. Blood specimens were taken from subjects after their Influenza infection was confirmed and also 2 weeks later. Our analysis only included samples from the acute phase (first timepoint). Subject samples were analyzed for cytokines, chemokines, growth factors and other mediators using bead-based Luminex multiplex assays or ELISA technology. In a preliminary analysis of the cytokine profiles, we detected significant differences between the measurements in year 1 and 2 of the study. These were likely caused by two factors: (1) The sampling strategy was modified between the two study years; and (2) Different labs quantified cytokines in each study year (personal communication, Sook-San Wong). We therefore used year 2 data for generating cytokine modules, but did not include it in our association analyses with clinical phenotypes presented below. Additional information regarding the design, sampling and subjects in SHIVERS cohort can be found in Table 1.

### 2.2. Adjustment for Mean Cytokine Concentration

To obtain the relative concentration of cytokines with respect to the overall level of cytokine secretion within each subject, cytokine concentrations were adjusted as follows: for a given cytokine, the levels for all subjects were regressed against the mean cytokine concentration. The adjusted cytokine concentrations were defined as the residuals from the regression. Formally, the adjusted values represent the level of unexplained deviation of that cytokine, from the expected cytokine level, given the average cytokine level of the subject. The full adjustment procedure for each *Cytokine*_*j*_ is as follows:

1. Compute the mean cytokines level for each subject in the dataset. Construct a vector of the means (with the length of the sample size).
2. Construct a regression model for *Cytokine*_*j*_, such that the vector of the means is used as a predictor for *Cytokine*_*j*_'s level in the sample, defined as the response variable:
  $$\begin{array}{l} Cytokine_{j}=\beta_{0j}+\beta_{1j}\cdot Mean+\epsilon_{j}. \end{array}$$
3. For each sample compute the expected *Cytokine*_*j*_ level using the regression model defined in II. Calculate the residue of the regression as:
  $$\begin{array}{l} Cytokine_{j_{adjusted}}=Cytokine_{j}-Cytokine_{j_{expected}}. \end{array}$$

As shown in Fiore-Gartland et al. (51) and above, adjustment can reveal interesting information about the relative deviation in cytokine levels in different individuals, which cannot be observed when analyzing absolute cytokine concentrations.

We note that the CytoMod adjustment procedure utilizes the values of all cytokines in a given dataset and may therefore be sensitive to the specific cytokines that were measured. To quantify the sensitivity of the adjustment procedure to cytokine selection, we conducted the following analysis: Subsets of cytokines were randomly selected from the original set of 37 cytokines in the PICFLU dataset; their size ranged between 2 and 36 cytokines. For the size of 36 cytokines, 37 subsets of cytokines were drawn, each containing the entire set of cytokines except for a single cytokine that was left out in each. For each subset size between 2 and 35, 50 different subsets of cytokines were randomly drawn. For each subset the adjustment procedure was conducted over the selected subset and the Spearman correlation was computed between the adjusted cytokine values of this subset, and their corresponding adjusted values over the entire set of 37 cytokines. Figure S1 presents the average median correlation across all 50 subsets, where the median was computed for each subset across all cytokines tested. We found that when drawing subsets of more than 10 cytokines, the average correlation to the original adjusted dataset was >0.95. Furthermore, when drawing subsets of 25 cytokines, the average correlation was 0.9899. This suggests that our adjustment method is robust given a sufficiently large set of cytokines.

### 2.3. Clustering

CytoMod is a modular approach for cytokine analysis that clusters cytokines based on pairwise correlations, to both amplify the signal they share and aid in interpretation by grouping putatively co-signaling molecules. Cytokines are grouped using a hierarchical clustering technique which iteratively pairs cytokines (and groups of cytokines) with similar behavior to generate a series of nested clusters. The clustering hierarchy can be represented by a tree-like graph (dendrogram) in which branches indicate the similarity between the formed subgroups of cytokines. By slicing the tree at a certain level we can obtain a set of distinct clusters. The dendrogram allows to graphically portray the clusters hierarchy and visualize the structure and data distribution in a manner that is intuitive for both computational and non-computational practitioners (55, 56, 60).

In this study, cytokine measurements from each dataset were clustered independently of the others. Measurements of different compartment samples in the same study were clustered independently due to notable differences in signaling patterns as shown in two different studies (49, 51). Importantly, clustering is performed over cytokines and not over subjects, to obtain groups of cytokines with similar expression profiles across subjects. Clusters were formed based on the correlation of adjusted and absolute cytokine levels, separately. Complete-linkage agglomerative hierarchical clustering was used to group cytokines (variables) with the Pearson's correlation coefficient as the distance metric. Complete linkage, which joins subclusters iteratively based on the closest maximum distance between pairs of variables in the subclusters, was used because it tends to form compact clusters. Since the approach suffers from sensitivity to minor perturbations in the data (56), we employed a bootstrap clustering method that was previously applied to gene expression data (61) in order to increase cluster robustness. The bootstrapping includes repetition of the clustering procedure on multiple perturbed subsets of the data, each formed by randomly drawing subject samples (with replacement) from the dataset. We repeated the clustering procedure on subject-level bootstrapped datasets 1,000 times. We recorded the number of times that each pair of cytokines clustered together across these 1,000 runs. The final hierarchical clustering was performed on this matrix of reliability fractions. Conceptually this can be thought of as a bootstrap estimate of cluster membership, simulating the reliability of each pair of cytokines to belong to the same cluster in repeated experiments on perturbed data under the same conditions.

The number of clusters (K) for each dataset was determined using the Tibshirani “gap statistic” heuristic method (62), which computes the marginal decrease in intracluster distance (ICD) for different K values, compared to the expected decrease under a null reference distribution of the data, assumed to be comprised of a single cluster. The estimate of the optimal K is the K for which the ICD falls the farthest below the reference curve while also taking into account the estimated deviation of the sampling distribution and simulation error (denoted by S). K is chosen as the first K that satisfies

$$\begin{array}{l} Gap(K)\geq Gap(K+1)-S_{k+1}. \end{array}$$

In our implementation we chose to test K values between 1 and 11 and generate a reference dataset by shuffling each feature (cytokine) independently of the others with 200 repetitions. For both real and null data distances between cytokines were defined using Pearson's correlation coefficient. For the real dataset bootstrapped clustering was performed as described above. To constrain the number of modules to be smaller than 6, and at least 2, in cases where the estimated best K found was not in these bounds or the condition was not satisfied for all K between 1 and 11, we chose K between 2 and 6 for which

$$\begin{array}{l} \underset{K}{\text{max }}Gap(K)-(Gap(K+1)-S_{k+1}). \end{array}$$

We chose to limit the number of clusters to 6 in order to reduce the formation of small (and possibly singleton) clusters. This threshold also affects the increase in statistical power for detecting associations, since as the number of clusters grows, more hypotheses will be tested and the adjusted *p*-values will decrease accordingly.

Finally, each cluster was used to calculate module scores for each subject in each dataset. Module scores were computed as the mean value of all cytokines that belong to the module after standardizing cytokine values to mean zero and unit variance.

### 2.4. Associations With Clinical Phenotypes

The primary analysis of cytokine modules included tests for associations with the clinical measures of disease severity available for each dataset using regression. All non-binary input and output variables were mean centered and variance scaled to unit variance. Logistic regression was used for all binary response variables and strength of effect was defined by an odds-ratio per unit increase in log-cytokine titer. For continuous response variables we used linear regression and strength of effect was defined using the log-cytokine regression coefficient (beta). Regression models controlled for the effects of variables that were previously used in each of the studies (49, 51, 58), as detailed in section 3.3. *P*-values for the coefficients describing the associations of cytokines and symptom scores were adjusted for multiple hypothesis tests within each cohort, compartment and adjustment method. *P*-values for the coefficients between module scores and symptom scores were also adjusted, separately from the cytokine coefficients. We report associations using two types of multiplicity adjustment methods: (1) false-discovery rate (FDR) using the Benjamini Hochberg procedure (63); (2) Family-wise error rate (FWER) using the Bonferroni-Holm method (64). Only associations with FDR-adjusted *q* ≤ 0.2 are shown. Associations that were significant using the more stringent FWER-adjusted *p*-value were marked using asterisks in each figure. All of the associations discussed below were FWER significant.

### 2.5. Defining Cytokine Cores

Cytokine measurements from each dataset were clustered into modules as described in section 2.3. Since airway samples were available only for two out of three studies, clustering comparison was only performed for the blood samples results. Comparison was performed for the absolute and adjusted clusters separately. For each we recorded the number of times each pair of cytokines clustered together in all three blood datasets. Cytokine cores were defined as groups of cytokines that clustered together across all three datasets. It should be noted that these cores may be refined when additional cytokine profile datasets are available.

Cytokine cores associations with phenotypes were calculated as described in section 2.4. A subject's score for each core was calculated based on the mean cytokine concentration of cytokines within the core, after standardizing each cytokine to mean zero and unit variance. *P*-values for the coefficients describing the associations of cytokines and phenotype scores were adjusted for multiple hypothesis tests within each presented dataset separately. *P*-values for the regression coefficients calculated for the core scores were adjusted separately than the coefficients calculated for individual cytokines. Individual cytokine *p*-values were adjusted across all cytokines and not only for the cytokines included in the core cytokine set.

Finally, we calculated pairwise Pearson correlations between cytokine cores within each blood dataset, i.e., PICFLU, SHIVERS, FLU09 and FLU09-healthy. *P*-values for the correlation coefficients were adjusted for multiple hypothesis tests within each dataset. The correlations were presented alongside each other in order to highlight trends across all datasets.

## 3. Results

We applied CytoMod to cytokine profiles of three independent cohorts (see section 2.1 for details) of consented subjects naturally infected with influenza virus: (1) PICFLU—a prospective multi-center study of children admitted to intensive care units with severe influenza virus infection (51); (2) FLU09—a prospective study of children presenting to the emergency room with influenza like-illness and their household members (49); and (3) Southern Hemisphere Influenza and Vaccine Effectiveness Research and Surveillance (SHIVERS)—a prospective study of influenza virus infected New Zealanders (58). The cohorts included 221, 161, and 87 subjects, respectively, who all tested positive for influenza. The FLU09 study provided an additional cohort of 142 healthy control volunteers that were not included in the main analyses and were analyzed separately in section 3.5.

To allow a direct comparison between the different cohorts, we limited our analysis to 37 cytokines that were measured from the blood of subjects in all three studies. These cytokines were also used to profile nasal wash from FLU09 subjects and endotracheal aspirates of PICFLU subjects. Cytokine concentrations (pg/mL) and subject ages were log-transformed for all analyses. Cytokine measurements from each study were analyzed independently of the others due to differences in subject characteristics and measurement methods. Measurements from different compartments (e.g., blood, nasal) were also analyzed separately due to notable differences in signaling patterns, as shown previously (49, 51). In total, five datasets were analyzed: FLU09 plasma, FLU09 nasal wash, PICFLU serum, PICFLU endotracheal aspirates and SHIVERS serum. We also analyzed an additional dataset of healthy controls that were sampled in the FLU09 study (49).

### 3.1. Generating Cytokine Modules

**Figure 2:**
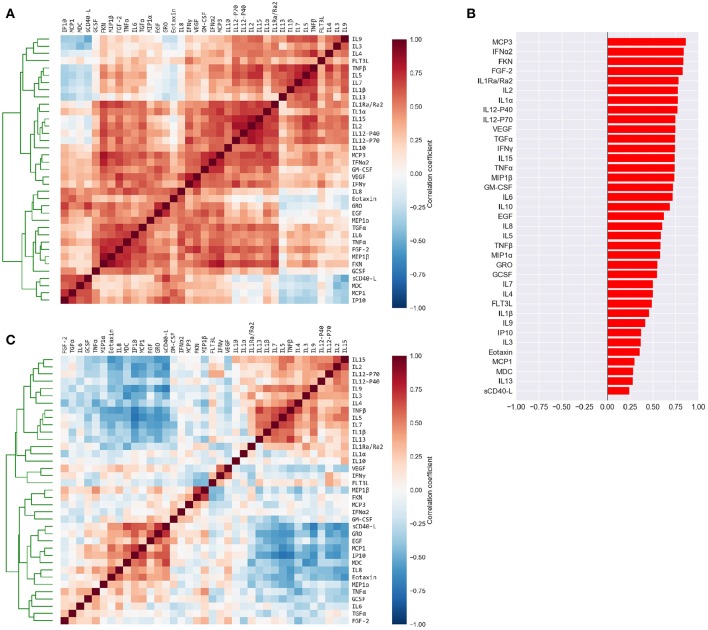
Cytokine levels are highly correlated to each other and to the mean cytokine level of each subject. **(A)** Pairwise Pearson's correlations among the absolute plasma cytokine levels in the FLU09 cohort. Cytokines were sorted along both axes using hierarchical clustering (complete-linkage). **(B)** Correlations between cytokine levels and mean cytokine levels for each subject. **(C)** Pairwise Pearson's correlations between cytokines following adjustment to the mean cytokine level (see Methods for details). Cytokines were sorted along both axes using complete-linkage.

**Figure 3:**
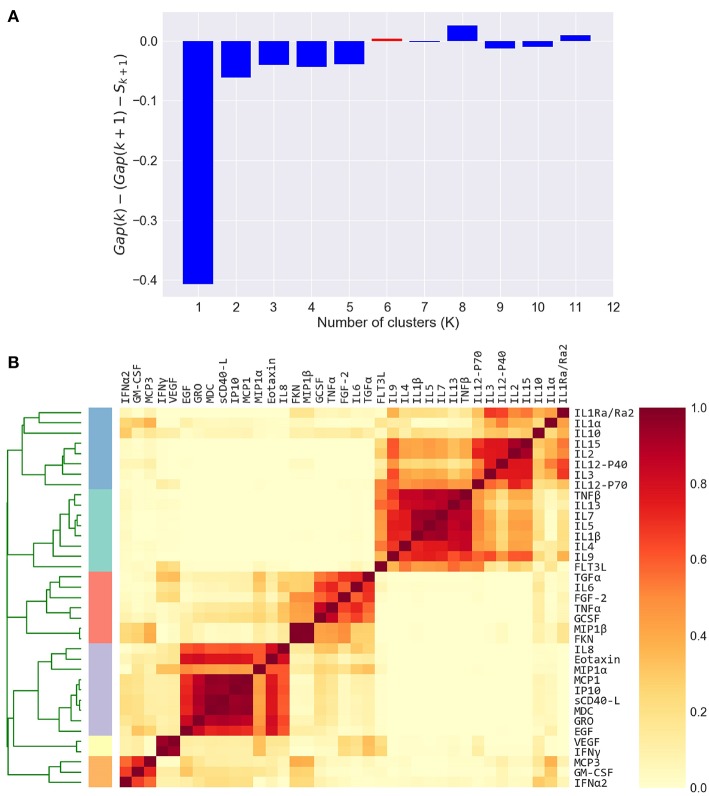
Defining cytokine modules on the FLU09 adjusted plasma cytokine profiles. **(A)** Automated selection of the optimal number of modules. The Tibshirani gap statistic is used to automatically determine the optimal number of modules. The cytokine profiles are clustered into *K* = 1−11 clusters and the optimal *K* is selected. The plot shows the δ gap statistic, defined as *Gap*(*K*) − (*Gap*(*K* + 1) − *S*_*k* + 1_) for *K* = 1−11. The optimal number of modules (*K*=6) is selected by identifying the first value of *K* for which this measure is positive, while also constraining it to vary between 2 and 6. **(B)** Heatmap of cytokine modules - Complete linkage clustering over the Pearson pairwise correlation similarity measure is used to cluster cytokines into *K* modules, where *K* is decided using the gap statistic. A clustering reliability score is computed over 1, 000 samplings of subjects that are sampled with replacement. The score for each pair of cytokines represents the fraction of times they clustered together across 1, 000 random samples. The reliability score of *K* = 6 is presented here. The final modules are then constructed by clustering the pairwise reliability scores, and are represented by the colored stripes below the clustering dendrogram.

To capture the underlying correlation structure induced by co-signaling cytokines, we developed a clustering-based approach to group cytokines into data-driven modules. Each module, represents a group of cytokines that co-vary across individuals within a given cohort. Modules are therefore defined separately for each cytokine dataset. The similarity between each pair of cytokines is defined by their Pearson correlation coefficient across all subjects, within a cohort. The similarity matrix is computed separately for each compartment, based on previous observations that found relatively low levels of correlations between cytokines across compartments as compared to within compartment similarities (49, 51). We computed the cytokine pairwise similarity matrices for each of the five datasets used in this study as outlined above (Figure 2A and Figures S2A, S3A, S4A, S5A). To define cytokine modules, we used an unsupervised hierarchical clustering algorithm that groups cytokines based on their pairwise similarity. Importantly, the algorithm does not incorporate any information regarding clinical phenotypes (i.e., clusters are not defined based on outcomes). The number of clusters was automatically selected. Specifically, we used complete-linkage agglomerative hierarchical clustering and the number of clusters (*K*) for each dataset was determined using the Tibshirani “gap statistic” method (62) (Figure 3A and Figures S6A–C), which selects the number of clusters based on the marginal decrease of within-cluster distances (see methods). Since minor perturbations of the data could affect the clusters obtained, a reliability score over each pair of cytokines was defined by computing the fraction of times a pair of cytokines were assigned to the same cluster over 1,000 randomly perturbed datasets (Figure 3B; see section 2.3). The final cytokine modules were defined over this pairwise reliability matrix. Cytokine values within each module were standardized (zero mean and unit variance) to ensure that each was given equal weight within a module. Given a set of cytokine modules, a subject-specific score was computed for every module defined by the mean cytokine concentration of all cytokines in the module, cytokine modules were subsequently used to detect associations between cytokine concentrations and clinical phenotypes.

### 3.2. High Correlation Among Cytokines Motivated Adjustment for Mean Concentration

The high positive correlation among the majority of cytokines in each compartment (or dataset) was also reflected in the significant positive correlations between each cytokine and the mean cytokine level within each subject (51) (Figure 2B and Figures S2B, S3B, S4B, S5B). Thus, subjects with a high concentration of one cytokine were relatively likely to have high concentrations of most of the other cytokines. We hypothesized that overall levels of immune activation (e.g., absolute number of immune cells in the blood) drive absolute cytokine concentrations. A high level of immune activation could therefore obscure cytokines expressed at relatively low levels. Furthermore, the absolute cytokine concentration could also be affected by technical artifacts such as sampling variability introduced by sample collection methods. Therefore, we developed an approach for adjusting cytokine measurements for the mean level within each sample using regression (detailed in section 2.2). An adjusted cytokine measurement reflects the level of unexplained deviation of that cytokine in a specific sample, from the expected cytokine level according to its association with the mean estimated across all samples. Correlations among cytokines after the adjustment can be substantially different, revealing associations that were previously obscured by the strong correlation with the mean (Figure 2C and Figures S2C, S3C, S4C, S5C). Therefore, following our previous work modules were constructed and analyzed using both absolute and adjusted cytokine concentrations separately for each dataset (51).

### 3.3. Modules Based on Absolute Cytokine Levels Were Associated With Influenza Clinical Phenotypes in Two Cohorts

For each study, we evaluated the association between each absolute cytokine module and the relevant clinical phenotypes recorded in the study, using linear or logistic regression models (detailed in section 2.4). Regression models controlled for the effects of age and other variables, as previously chosen for each of the three cohorts (49, 51, 58). For purposes of comparison, we also evaluated the association of each absolute individual cytokine with the phenotypes. *P*-values for the coefficients describing the associations of modules and cytokines with clinical phenotypes were adjusted for multiple hypothesis tests within each figure presented (i.e., across cytokines or cytokine modules, but within cohort, compartment and absolute/adjusted module set). The *P*-values for the module and cytokine coefficients were adjusted independently. Family-wise error rate (FWER)-adjusted *p*-values using the Bonferroni-Holm method (64) were calculated and are presented in each figure using asterisks. Only associations with a false-discovery rate (FDR)-adjusted *q* ≤ 0.2 are shown [using the Benjamini' Hochberg procedure (63)]. However, only associations with FWER-p ≤ 0.05 were considered statistically significant.

**Figure 4:**
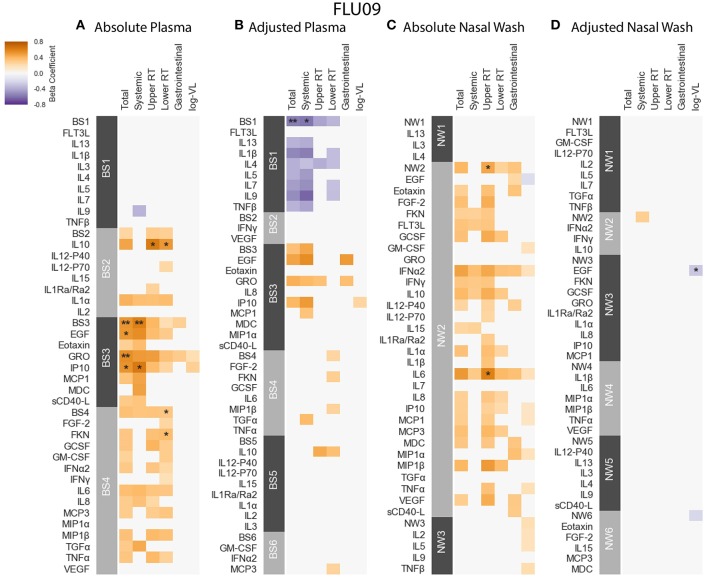
FLU09 cytokine associations with clinical phenotypes. Associations were identified using linear regression controlling for patients age using both absolute and adjusted plasma samples **(A,B)**, and absolute and adjusted nasal wash samples **(C,D)**. Modules of covarying cytokines were constructed separately for absolute and adjusted cytokine measurements from plasma or nasal wash samples. We then tested associations with several clincal phenotypes described in section 2.1: upper respiratory tract (URT) symptoms, lower respiratory tract (LRT) symptoms, systemic symptoms, gastrointestinal symptoms and log viral load (VL). Each cytokine or module is indicated along the rows, grouped by their assigned module. Heatmap color indicates the direction and magnitude of the regression coefficient between cytokine or module level with a given clinical phenotype. Only associations with false-discovery rate (FDR)-adjusted *q*-value ≤ 0.2 are colored. Asterisks indicate family-wise error rate (FWER)-adjusted *p*-values with ***, **, and * indicating *p* ≤ 0.0005, 0.005, and 0.05, respectively.

**FLU09** - The associations with clinical phenotypes in influenza-positive FLU09 absolute datasets were calculated using linear regression adjusted for age (Figures 4A,C and Tables S1, S3). Modules and cytokines were tested for associations with several clinical phenotype groups recorded in the study (detailed in section 2.1): upper respiratory tract (Upper RT) symptoms, lower respiratory tract (Lower RT) symptoms, systemic symptoms, gastrointestinal symptoms, total symptoms and (log) viral-load (log-VL). Significant positive associations were observed with the absolute plasma modules. For example, absolute Blood Sample 3 module (BS3) was positively associated with total and systemic symptoms, and absolute BS4 was associated with lower RT symptoms (regression coefficients of 0.529, 0.605, 0.322, and FWER *p*-values of 0.0035, 0.0037, 0.0304, respectively). Individual cytokines within these modules were also significantly associated as follows: BS3 cytokines EGF, GRO and IP-10 positively associated with total symptoms and IP-10 also associated with systemic symptoms; the BS4 cytokine Fractalkine (FKN) was positively associated with lower RT symptoms. While most of the regression coefficients of these cytokines were slightly higher than those of their modules, the statistical significance of the absolute associations after FWER adjustment was stronger for the modules than the individual cytokines in 4 of 5 (significant) cases; only the significance of the Fractalkine association was stronger than that of the BS4 module to which it belongs. In addition, IL-10 was significantly associated with both upper and lower RT, while the BS2 module to which it belonged was not significantly associated with any symptoms. This increase in statistical significance is directly attributable to the reduction in the number of statistical tests across which multiplicity adjustment is applied. In the absolute-module set analysis of the nasal wash samples, the majority of cytokines clustered together into one module (NW2), perhaps due to high immune activation at the site of infection. Absolute NW2 was significantly positively associated with upper RT symptoms (regression coefficient 0.46, FWER *p* = 0.029), however, NW2 cytokine IL-6 had a stronger significant positive association with the same phenotypes. It should be noted that all of the previously reported cytokine associations with symptom scores identified using data from years 1 to 2 of the FLU09 study (using an FDR threshold of 0.2) (49), were re-confirmed in our current analysis using the complete cohort from years 1 to 5 (Figure S9), and additional associations were found in the current analysis.

**Figure 5:**
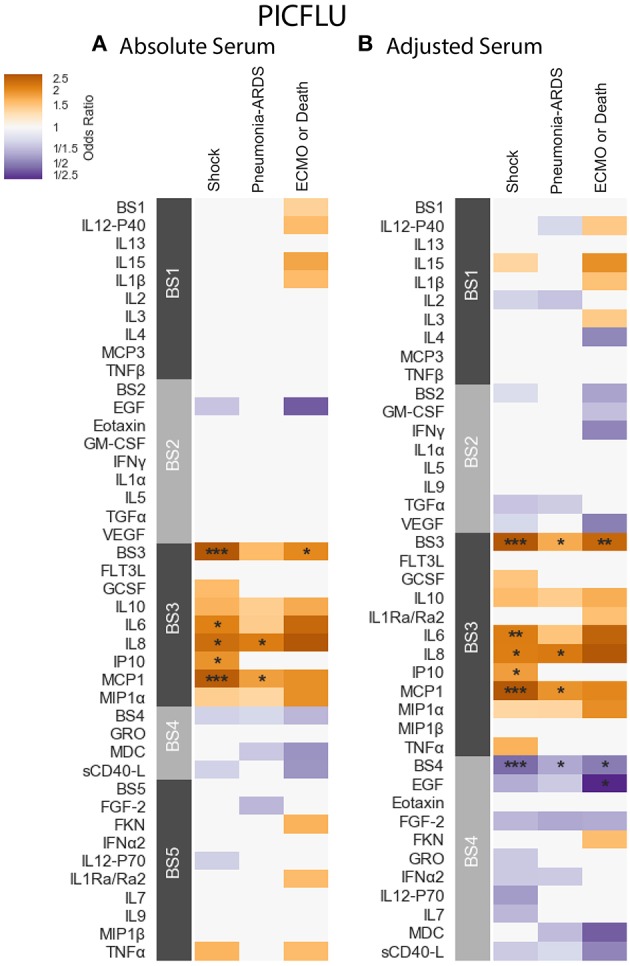
PICFLU serum cytokine associations with clinical phenotypes identified using logistic regression while controlling for patients age and bacterial coinfection. Modules constructed of covarying cytokines [absolute **(A)** and adjusted **(B)** measurements separately] from serum samples, were tested for associations with the clinical phenotypes described in section 2.1: shock, pneumonia-ARDS and ECMO or death. Each cytokine or module is indicated along the rows, grouped by their assigned module. Heatmap color indicates the direction and magnitude of the regression coefficient between cytokine or module level with a given clinical phenotype with and without the complication. Only associations with false-discovery rate (FDR)-adjusted *q* ≤ 0.2 are colored. Asterisks indicate family-wise error rate (FWER)-adjusted *p*-values with ***, **, and * indicating *p* ≤ 0.0005, 0.005, and 0.05, respectively.

**PICFLU** - Positive significant correlations were also observed in the absolute PICFLU serum associations with clinical phenotypes portrayed in Figure 5A and Table S5. These associations, calculated using logistic regression, were adjusted for age and bacterial coinfection (see section 2.1 for details). The absolute BS3 module was positively associated with both shock and ECMO or death (odds ratio 2.75, 2.04, FWER *p*-values 0.00002, 0.0286, respectively). The BS3 cytokines IL-6 and IP-10 had an association with shock, while IL-8 and MCP-1 had an association with both shock and pneumonia-ARDS. The BS3 association with ECMO or death was significant while none of the individual cytokines in the module were significantly associated. The strength of the association with shock for all individual BS3 cytokines was weaker than for the module as a whole. On the contrary, absolute IL-8 and MCP-1 had a significant association with pneumonia-ARDS, while the absolute BS3 module did not. The PICFLU absolute endotracheal samples did not have any significant associations with outcomes (FWER-p > 0.05; Figure S7A and Table S7).

**SHIVERS** - Due to differences in sampling strategy during the first and second years of the study, associations with phenotype were calculated only for subjects from the first year of the SHIVERS study (*n* = 52). Logistic Regression models included adjustment for age, ethnicity and sampling time. No significant associations were detected among absolute individual serum cytokines, with severe acute respiratory illness (SARI) (Figure S8A and Table S9). However, we note that univariate associations previously reported for this cohort (58), which were not adjusted for multiplicity testing across cytokines, were in overall agreement with the cytokine associations reported here for FLU09. In particular, EGF, GRO, sCD40-L and MCP-1, all clustered together in SHIVERS to absolute module BS3 and positively correlated with SARI in the SHIVERS previous analysis. In our current analysis of FLU09 these cytokines belong to the absolute BS3 module, which was positively associated with total and systemic symptom scores. In addition, Fractalkine, VEGF, TNF-α and GCSF belong to the BS4 absolute module in FLU09 which was positively associated with lower respiratory tract symptom scores (LRT). They were also previously reported to be positively associated with SARI in SHIVERS. In particular, Fractalkine was also significantly associated with LRT scores In FLU09 and had an odds-ratio of 16.52 for SARI in SHIVERS (58).

### 3.4. Adjustment for Mean Cytokine Level Reveals Negative Associations Between Modules and Clinical Phenotypes

While none of the absolute concentrations of cytokines or their modules were negatively associated with clinical phenotypes, we found several significant negative associations using cytokines and cytokine modules that had been adjusted for the mean cytokine concentration. Interestingly, some, but not all of the significant positive associations that were identified using absolute cytokine concentrations were also significant after adjustment for the mean.

**FLU09** - as seen in Figure 4B, the adjusted BS1 module containing FLT3L, IL-13, IL-1β, IL-4, IL-5, IL-7, IL-9, and TNF-β was found to be significantly negatively associated with total and systemic symptoms (regression coefficients –0.557, –0.582, FWER *p*-values 0.0011, 0.008, respectively, as detailed in Table S2). Individual cytokines in this module were predominantly negatively associated with symptom scores, some with FDR ≤ 0.2, however, none of these associations were significant after FWER adjustment. The adjusted nasal wash modules did not have any significant positive associations with symptom scores (Figure 4D and Table S4). A single significant negative association was found between adjusted EGF concentrations and viral load (regression coefficient -0.281, FWER-p = 0.0157).

**PICFLU** - The adjusted BS4 module containing EGF, Eotaxin, FGF-2, Fractalkine (FKN), GRO, IFN-α2, IL-12-P70, IL-7, MDC, and sCD40-L was negatively associated with shock, pneumonia-ARDS and ECMO or death (odds ratio 0.463, 0.598, 0.494, FWER-p = 0.0002, 0.0155, 0.0283, respectively; Table S6). The adjusted concentration of EGF (member of BS4) was also found to be negatively associated with ECMO or death (OR = 0.211, FWER-p 0.043), albeit more weakly that of the BS4 module. No other individual adjusted cytokines were found to be negatively associated with clinical phenotypes. The adjusted BS3 module, which contained a similar group of cytokines to that of the absolute BS3 module, had positive associations with shock, pneumonia-ARDS and ECMO or death (odds ratio 3.01, 1.75, 2.46, FWER *p*-values 0.000002, 0.0095, 0.0042, respectively). The BS3 adjusted cytokines IL-6 and IP-10 were associated with shock, while IL-8 and MCP-1 were associated with both shock and pneumonia-ARDS. As with the absolute cytokine analysis, adjusted BS3 was associated with ECMO or death, while none of its constituents were associated on their own. The significance of all adjusted BS3 member cytokines with shock was weaker than the module's; the significance of the adjusted IL-8 association with pneumonia-ARDS was also weaker than that of BS3. However, the significance of the association of adjusted MCP-1 with pneumonia-ARDS was stronger than that of its module BS3.

**SHIVERS** - While no significant associations were detected among adjusted individual serum cytokines, we found that the adjusted BS6 module was positively associated with severe acute respiratory illness (SARI) (Figure S8B and Table S10). Furthermore, IL-4, IL-13, and TNF-β were part of the adjusted BS1 module of SHIVERS and were also in the adjusted BS1 module of FLU09 that was negatively associated with total and systemic symptom scores, as well as negatively associated with SARI in the previous report on SHIVERS (58).

### 3.5. Subsets of Cytokine Clusters Were Similar Across Datasets

**Figure 6:**
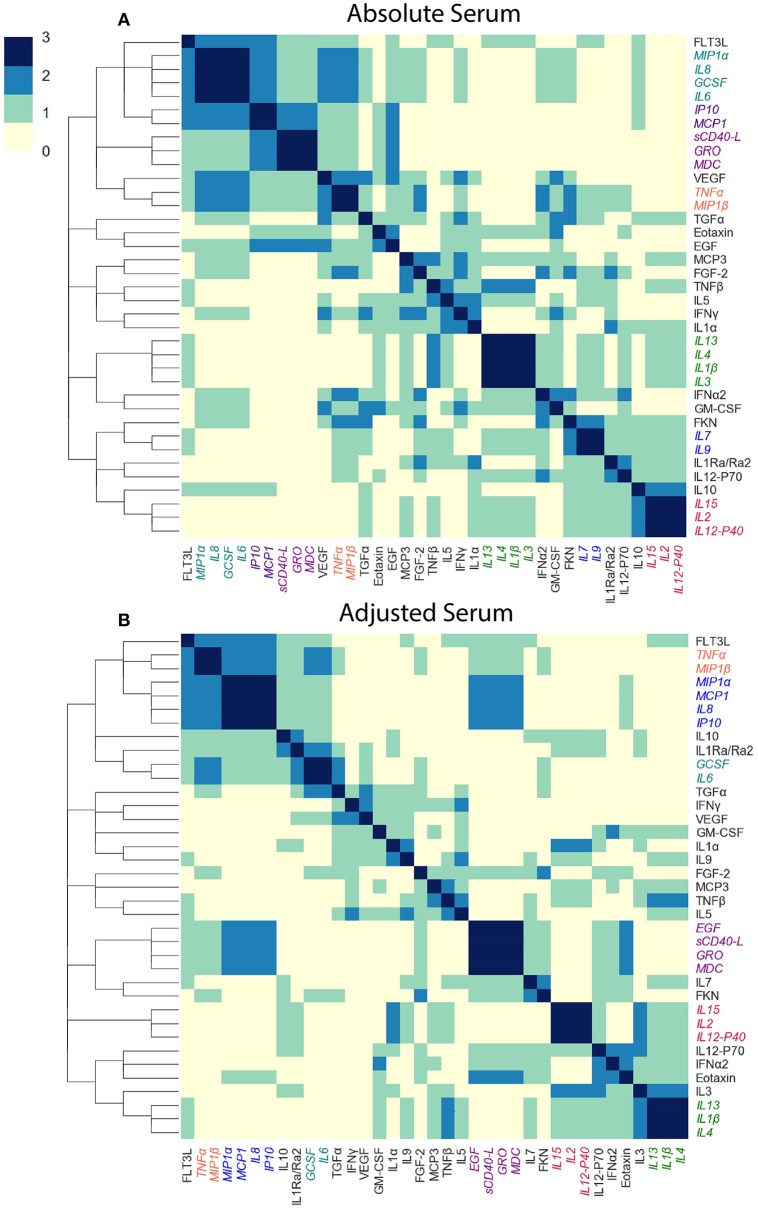
Defining cytokine cores. By leveraging information across cytokine profile datasets, we can identify cytokine cores—subsets of cytokines that consistently co-signal across all three blood datasets used in this study. Heatmaps of the number of times each pair of cytokines clustered together in all three cohorts, for adjusted **(A)** and absolute **(B)** blood sample data independently. Cytokine names are colored by cytokine cores.

We next asked whether cytokine modules were consistent across datasets, i.e., were there cytokine “cores”—clusters of cytokines that were consistently correlated during influenza infection. Since airway samples were available only for two out of three cohorts, this analysis was only performed using blood sample (serum or plasma) modules. To identify cytokine cores, we tallied the number of times that each pair of cytokines clustered together across the three blood datasets (Figures 6A,B). Cytokine cores were defined as groups of cytokines that clustered together in all three datasets. Cytokine cores were defined separately for the absolute and adjusted cytokine modules (Table 2). There is overall agreement between the absolute and adjusted cytokine cores. The most striking difference is the division of IP-10, MCP-1, IL-8, and MIP-1α into two different subsets.

**Table 2:** Cytokine cores identified in absolute and adjusted blood samples independently.

**Adjusted blood cores**	**Absolute blood cores**
(1) GRO, MDC, sCD40-L, EGF	(1) GRO, MDC, sCD40-L
(2) IL-2, IL12-P40, IL-15	(2) IL-2, IL-12-P40, IL-15
(3) IL-1β, IL-4, IL-13	(3) IL-1β, IL-4, IL-13, IL-3
(4) MIP1-β, TNF-α	(4) MIP1-β, TNF-α
(5) IP-10, MCP-1, IL-8, MIP1-α	(5) IP-10, MCP-1
(6) GCSF, IL-6	(6) GCSF, IL-6, IL-8, MIP-1α
	(7) IL-7, IL-9

To determine whether the cytokine cores were unique to influenza infected subjects, we constructed modules of adjusted and absolute plasma samples provided by 142 healthy volunteers in the FLU09 study. We found that overall, cytokine cores were consistent across influenza-infected and healthy controls with two exceptions: in the absolute cores, GCSF did not cluster together with other core-6 cytokines and cores-4 and -6 were not identified in the adjusted modules of healthy controls.

### 3.6. Core Modules Were Also Associated With Clinical Phenotypes

Each absolute or adjusted core was composed of cytokines that clustered together into the same module in all three cohorts (Tables 3, 4). For example, adjusted core-2 was composed of IL-12-P40, IL-15 and IL-2, which were members of PICFLU adjusted BS1, FLU09 adjusted BS5 and also SHIVERS adjusted BS5. We noted that most adjusted and some of the absolute cores (Figure 6) were composed of cytokines that were members of modules that exhibited strong associations with clinical phenotypes. For example, adjusted core-1 contained GRO, MDC, sCD40-L, and EGF, members of the PICFLU adjusted module BS4 (Figure 5B), which was negatively correlated with poor clinical phenotypes. Surprisingly, they were also members of the adjusted module BS3 from FLU09 (Figure 4B), which in contrast had mostly positive associations (significant only in the absolute measurements). Adjusted core-3 contained IL-1β, IL-4, and IL-13 that were part of the FLU09 adjusted module BS1, which was negatively associated with several symptom scores. Adjusted core-5 contained IP-10, MCP-1, IL-8, and MIP-1α that were part of FLU09 adjusted BS3 mentioned above, and also of adjusted PICFLU module BS3 which had significant positive associations with all phenotypes. Absolute core-5 and absolute core-6 cytokines were part of FLU09 and PICFLU absolute modules that had significant positive associations with phenotypes (Figures 4A, 5A).

**Table 3:** Modules that construct the absolute cytokine cores by dataset.

**Absolute core**	**PICFLU**	**FLU09**	**SHIVERS**
(1)	BS4	BS3	BS3
(2)	BS1	BS2	BS2
(3)	BS1	BS1	BS1
(4)	BS5	BS4	BS3
(5)	BS3	BS3	BS3
(6)	BS3	BS4	BS3
(7)	BS5	BS1	BS2

**Table 4:** Modules that construct the adjusted cytokine cores by dataset.

**Adjusted core**	**PICFLU**	**FLU09**	**SHIVERS**
(1)	BS4	BS3	BS3
(2)	BS1	BS5	BS5
(3)	BS1	BS1	BS1
(4)	BS3	BS4	BS3
(5)	BS3	BS3	BS3
(6)	BS3	BS4	BS4

**Figure 7:**
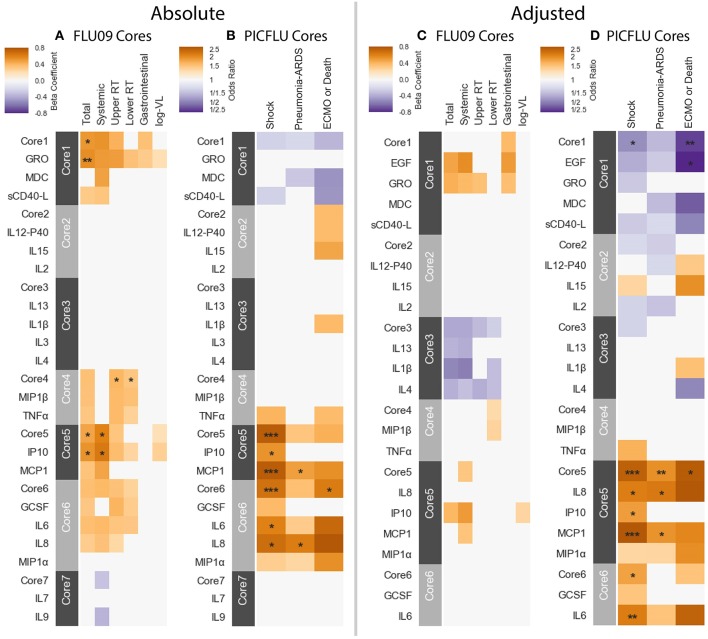
Cores constructed from groups of covarying cytokines (absolute and adjusted measurements separately) as detailed in section 3.5. Associations between cytokine cores and clinical phenotypes are shown for FLU09 **(A,C)** and PICFLU **(B,D)** for both raw and adjusted cytokine levels. Blood cytokine cores associations with phenotypes were estimated using regression while also controlling for other variables as described in section 3.3. Each cytokine or core is indicated along the rows. Heatmap color indicates the direction and magnitude of the regression coefficient. For each individual cytokine FDR and FWER adjustments are shown are controlled over all 37 cytokines. Only associations with false-discovery rate (FDR)-adjusted *q* ≤ 0.2 are colored. Asterisks indicate family-wise error rate (FWER)-adjusted *p*-values with ***, **, and * indicating *p* ≤ 0.0005, 0.005, and 0.05, respectively.

We then tested for associations between the cytokine cores and clinical phenotypes, using the same methodology described above. A subject's score for each core was calculated based on the mean cytokine concentration of cytokines within the core, after standardizing each cytokine to mean zero and unit variance. Figure 7 portrays the associations to clinical outcomes and symptoms for absolute and adjusted blood cytokines of influenza-positive FLU09 and PICFLU subjects, respectively. *P*-values for the coefficients describing the associations of cytokines and symptom scores were adjusted for multiple hypothesis tests within each presented dataset separately. *P*-values for the regression coefficients calculated for the core scores were adjusted independently of the coefficients calculated for individual cytokines. Individual cytokine *p*-values were adjusted across all cytokines and not only for the cytokines included in the core cytokine set.

**FLU09** - None of the adjusted plasma FLU09 cores were significantly associated with clinical symptoms, but trends were in agreement with the module associations (Figure 7 and Tables S11, S12). However, absolute cores were associated with symptoms: absolute core-1 was associated with total symptoms; core-4 was associated with lower and upper RT symptoms; and core-5 was associated with total and systemic symptoms. Each core's corresponding module was similarly associated with symptoms: BS3 which contained absolute core-1 and core-5 cytokines was associated with total and systemic symptoms; BS4 which contained absolute core-4 cytokines was associated with lower RT symptoms but not with upper RT symptoms (while core-4 itself was associated with both).

**PICFLU** - Significant associations were found with both absolute and adjusted cores (Figure 7 and Tables S13, S14). Absolute core-5 was positively associated with shock, absolute core-6 was positively correlated with shock and ECMO or death. Absolute core-5 and core-6 cytokines were members of absolute module BS3, which was also positively associated with shock and ECMO or death. Three adjusted cores, originating in two different modules, were associated with outcomes: adjusted core-1 was negatively associated with shock and ECMO or death, adjusted core-5 was positively associated with shock, pneumonia-ARDS and ECMO or death, and adjusted core-6 was positively associated with shock. Adjusted BS4 which contained adjusted core-1 cytokines was associated with all outcomes, while core-1 was negatively, but not significantly associated with pneumonia-ARDS after FWER adjustment. Adjusted BS3 which contained adjusted core-5 and -6 cytokines was positively associated with all outcomes.

**SHIVERS** - In the SHIVERS cohort, neither the absolute or adjusted cores were significantly associated with the SARI phenotype. The lack of association may be due in part to the small sample size in the first year of the study (*n* = 52).

### 3.7. Correlations Among Core Modulations Were Consistent Across Cohorts

**Figure 8:**
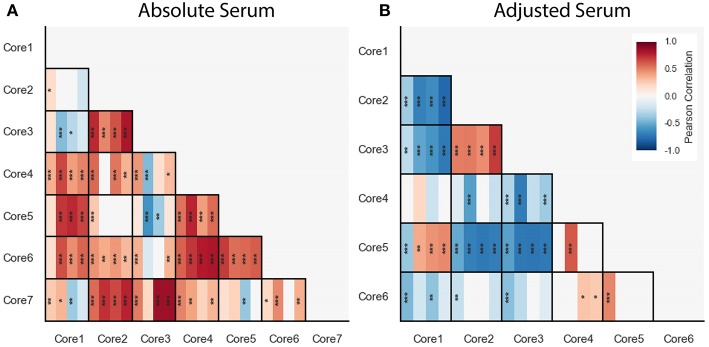
Pairwise Pearson correlations between absolute **(A)** and adjusted **(B)** blood cytokine cores within each dataset, presented with vertical stripes from left to right: PICFLU, SHIVERS, FLU09, and FLU09-healthy. Heatmap color indicates the correlation coefficient. *P*-values for the coefficients were adjusted for multiple hypothesis tests within each dataset separately. Only associations with false-discovery rate (FDR)-adjusted *q* ≤ 0.2 are colored. Asterisks indicate family-wise error rate (FWER)-adjusted *p*-values with ***, **, and * indicating *p* ≤ 0.0005, 0.005, and 0.05, respectively.

We computed correlations between cores within each of the blood cytokine profile datasets, including the FLU09 healthy controls (see section 2.5 for details). Overall we found mostly positive significant correlations between absolute cores that were consistent across all datasets, with a few notable exceptions: cores-1 and -7 and cores-3 and -4 (Figure 8A).

We also computed pairwise correlations for the adjusted cores (Figure 8B). Similarly to the absolute cores, overall correlations between cores were consistent across datasets except for one pair of cores (core-1 and core-5).

## 4. Discussion

Here we presented CytoMod—a data driven approach for analyzing cytokine profiles and their association with clinical phenotypes. Our approach leverages the inherent redundancy of cytokines to form modules—clusters of cytokines whose signals correlate across a cohort of individuals. CytoMod is an unsupervised method—i.e., it does not use any information about clinical phenotypes or outcomes to identify cytokine modules. Using cytokine modules increases the statistical power to detect associations with clinical phenotypes, by amplifying the signal within a module relative to the noise, as well as reducing the number of tests subject to multiplicity adjustment. It also allows for the identification of data-specific co-signaling cytokines, which may provide clues about the underlying immunological pathways. A preliminary version of CytoMod was applied in the analysis of the PICFLU cohort (51). Importantly, the method presented here includes automated selection of the number of modules using the gap-statistic heuristic. Indeed, when applied to the PICFLU cohort, it identified different numbers of modules than those analyzed in our previous work.

To our knowledge CytoMod is the first method for the analysis of cytokine profiles and clinical phenotypes that utilizes modules identified within cytokine expression data. In the age of multi-omics approaches, novel strategies for the integration of multiplex data with clinical outcome information can assist in the identification of complex pathological alterations of physiological networks. CytoMod only requires a dataset of cytokine measurements and (optionally) clinical phenotypes. Importantly, it does not assume that modules necessarily capture biological function.

CytoMod is based on unsupervised clustering which can help uncover inherent structures within a given dataset. Our work is related to previous work on methods for cluster analysis of variables (65–67), which groups together variables which are strongly related to each other and hold similar information. CytoMod can also be viewed as a dimensionality reduction method for cytokine profiles. There are a variety of other methods for dimensionality reduction that have been widely used for visualization and analysis of biological data. These include methods such as Principal Components Analysis (PCA) (68, 69), Linear Discriminant Analysis (LDA) (70), Factor Analysis (71) and t-sne (72). Most of these methods project the samples into a low-dimensional space by creating new features from linear combinations of the original features. In this new space the original coordinates (or features/cytokines) are not retained, thereby reducing the ability to draw biological interpretation. In contrast, our modules retain interpretability by grouping together individual cytokines that are co-expressed and can be further studied to allow gaining new insights into the underlying biological processes that generate these structures.

We applied CytoMod to three independent cohorts of influenza-infected subjects. The analyses of SHIVERS and FLU09 datasets presented here included previously unpublished data from additional study years, as well as data from healthy volunteers. To allow comparisons between the cohorts, we limited the number of cytokines analyzed to a subset of 37 cytokines that were quantified in all three cohorts. We found that in two of these cohorts, modules were significantly associated with clinical phenotypes, and in most cases the associations were stronger than those of individual cytokines within the module. Specifically, we found that across all modules in these two datasets, the association of the module with outcomes was more significant than that of an individual cytokine in 14 out of 22 cases in which the cytokine's association was significant. Furthermore, in 6 cases, a module had a significant association with a phenotype while non of its cytokines had any significant association.

In our previous analysis of FLU09, we analyzed only 11 pre-selected cytokines using data from years 1 to 2. In our current manuscript, we analyzed data from the entire study (years 1–5). We identified novel associations between modules and clinical phenotypes. Specifically, the adjusted BS1 module containing FLT3L, IL-13, IL-1β, IL-4, IL-5, IL-7, IL-9, and TNF-β was found to be significantly negatively associated with total and systemic symptoms. Out of these cytokines, only IL-1β was included in the previous analysis. In addition, we found novel associations of the absolute EGF, GRO, FKN levels that were not previously reported. In the analysis of the PICFLU study, which only included children admitted to the ICU with influenza infection, we found that the serum module BS3 is significantly associated with Shock and ECMO/death outcomes. Interestingly, this module contains IL-6, IL-8, and MCP-1, which have been previously reported to be hyperactivated in subjects with severe influenza infection (28). No significant associations with clinical phenotypes were detected in the SHIVERS cohort, though this may be due in part to its small sample size (*n* = 52), and sampling variability (58) which further limits the ability to detect associations. Interestingly, we note that univariate associations previously reported for this cohort (58), which were not adjusted for multiplicity testing across cytokines, were in overall agreement with the module associations reported here for FLU09 and in some cases with the cytokine cores.

We focused here on analyzing cytokine associations with outcomes that were significant following a stringent FWER adjustment procedure. In fact, all of the previously reported FDR-adjusted FLU09 associations based on data from years 1–2 (49) were also significant using FDR-adjustment on the complete years 1–5 dataset, and three of them were also FWER significant (when adjusted across all 37 cytokines analyzed here). These findings suggest that many of the associations with FDR *q*-values ≤ 0.2 may also be worth further exploration (Figure S9). The fact that many individual cytokines within FWER significant modules have associations with clinical phenotypes with the less stringent FDR *q*-value threshold of 0.2, while only a few of them have FWER significant associations, demonstrates the increase in statistical power provided by the modular cytokine approach. The CytoMod method considers both absolute and adjusted cytokine levels, since immune cells may be sensitive both to absolute and relative cytokine concentrations (73–75). While some positive associations with clinical phenotypes were observed using both the absolute and adjusted cytokines and modules, we found that significant negative associations with clinical phenotypes were found only with the adjusted modules. This is likely due to the fact that adjustment to the overall cytokine expression level may uncover differences in cytokine levels that are expressed at relatively low levels. Specifically, we found that the adjusted BS1 module in FLU09 was negatively associated with total and systemic symptoms, and that the adjusted BS4 module in PICFLU was negatively associated with the clinical phenotypes of shock, pneumonia-ARDS and ECMO-death. Interestingly, some of the BS4 cytokines were positively associated to FLU09 symptom scores when considering the less stringent FDR *q*-value ≤ 0.2. These results highlight the importance of analyzing both absolute and adjusted cytokine levels.

By analyzing three independent cohorts of subjects naturally infected with influenza, we were able to identify cytokine “cores”—subsets of cytokines that consistently clustered together across datasets. Cores were extracted from the modules directly and were identified without using any information about clinical outcomes or subject demographics. Interestingly, the majority of these cores clustered together in the set of 142 healthy controls from the FLU09 study, suggesting that these cores may represent sets of co-signaling cytokines. Some of these cores include cytokines that have been reported to have similar roles: For example: (1) adjusted core-3 which includes IL-1β, IL-4, and IL-13 contribute to epithelial repair mechanisms (4); (2) IP-10, MCP-1, IL-8, and MIP-1α which belong to adjusted core-5 are chemokines that are key inflammatory mediators (1); (3) IL-2, IL-15, and IL-12-p40 in adjusted and absolute core-2 are involved in T-cell activation (76, 77).

While we found that the cytokine cores were significantly associated with clinical phenotypes, the associations of the cytokine modules that were defined separately for each dataset were overall stronger. This is not surprising for two reasons: (1) using the strict definition of cores used here (co-clustering in all 3 datasets), cores are typically smaller than data-driven modules and are more sensitive to measurement noise; (2) Data driven modules of a specific cohort may also be affected by other covariates which may be specific to that cohort, and are not captured by the cytokine “cores” which are created using multiple datasets.

We analyzed correlations between cytokine cores, and compared these across datasets in both the absolute and adjusted datasets. We found that overall, the correlations between different cytokine cores were consistent across the three datasets, as well as in a cohort of healthy controls. However, we found one notable exception: adjusted core-1 and core-5 were negatively associated only in the PICFLU dataset. This is also reflected in the fact that core-1 (EGF, GRO, MDC, and sCD40-L) was weakly positively associated with outcome in a cohort of mild influenza infection (FLU09) and was negatively associated with outcome of severe influenza infection (PICFLU).

The existence of cytokine cores, and their association with clinical phenotypes despite a variety of differences between the cohorts suggest that these cores may represent stable underlying cytokine modules that consistently co-signal during influenza infection, and in some cases also in a healthy state. Cytokine cores may relate to specific functions and underlying biological processes that govern the complex cytokine signaling network. Nonetheless, defining robust cytokine cores requires large scale analysis of multiple cytokine datasets. As additional cytokine profile datasets are generated and made publicly available, cores can be dynamically re-defined, including defining “softer” probabilistic cores based on frequency of co-occurrence across many datasets and conditions. Identification of consistent cytokine subsets may provide a basis for the selection of biomarkers and the development of targeted immune assays, as part of a novel approach for developing future point-of-care diagnostic tests based on cytokine measurements that may be used for many different infections.

CytoMod groups cytokines into modules so that each cytokine belongs to a single module. We hypothesize that similar to genes, each cytokine may play several functional roles under different immune contexts. This would be best captured by “soft” modules, in which each cytokine may belong to more than one module. Once a sufficiently large number of cytokine datasets are analyzed such softer modules may be identified and annotated, similar to annotations of gene modules (52, 57). Our analysis of three datasets should be viewed as a first step in this direction.

CytoMod can be applied to any cytokine profile dataset and does not make any assumptions regarding the specific technology that was used to quantify cytokines. Furthermore, the modular approach allows identification of co-signaling cytokines across study years, even if the specific kit used to quantify cytokines was changed during the study, or other changes to the study were implemented. This is due to the fact that correlations are computed between cytokines across study subjects. Indeed despite significant differences between the cytokine measurements in years 1 and 2 of the SHIVERS study, we used both years to generate cytokine modules for this dataset.

In summary, using a modular approach to analyze cytokine profile datasets provides two major advantages: (1) It increases statistical power to detect associations with clinical phenotypes; and (2) By comparing modules obtained from different independently sampled datasets, we can identify cytokine cores - sets of consistently co-signaling cytokines. By aggregating cytokine information across datasets, this approach may help identify inherent, and condition-specific groupings of cytokines, providing the basis for future mechanistic molecular studies. A Python implementation code of CytoMod can be found at https://github.com/liel-cohen/CytoMod as well as in an interactive Jupyter Notebook available at https://nbviewer.jupyter.org/github/liel-cohen/CytoMod/blob/master/cytomod_notebook.ipynb.

## Data Availability

All datasets generated for this study are included in the Supplementary Files.

## Author Contributions

AF-G, LC, and TH developed the computational method. AF-G and LC analyzed data. TH designed the study. AR, AP-M, S-SW, JR, TW, RS, QH, RW, and PT enrolled the clinical cohorts, and generated data. LC and TH wrote the paper. AF-G, AR, S-SW, and PT commented and edited the paper.

### Conflict of Interest Statement

The authors declare that the research was conducted in the absence of any commercial or financial relationships that could be construed as a potential conflict of interest.
