# Supplementary material for: A Modular Cytokine Analysis Method Reveals Novel Associations With Clinical Phenotypes and Identifies Sets of Co-signaling Cytokines Across Influenza Natural Infection Cohorts and Healthy Controls
Source: Front Immunol. 2019 Jun 18;10:1338. doi: 10.3389/fimmu.2019.01338 (PMC6594355; doi:10.3389/fimmu.2019.01338)
Supplement: Supplementary file 4 [file Data_Sheet_1.pdf]

Supplementary Material:
A modular cytokine analysis method reveals novel associations with clinical phenotypes and identifies sets of co-signaling cytokines across influenza natural infection cohorts and healthy controls

1 SUPPLEMENTARY FIGURES AND TABLES

1.1 Figures

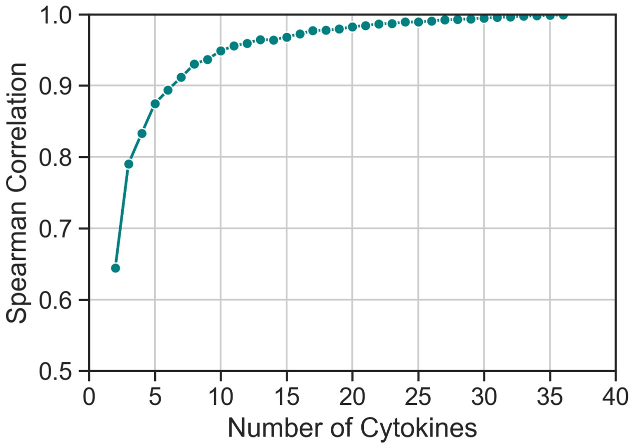

Figure S1. Subsets of cytokines were randomly selected from the original set of 37 cytokines in the PICFLU dataset; their size ranged between 2 and 36 cytokines. For the size of 36 cytokines, 37 subsets of cytokines were drawn, each containing the entire set of cytokines except for a single cytokine that was left out in each. For each subset size between 2 and 35, 50 different subsets of cytokines were randomly drawn. For each subset the adjustment procedure was conducted over the selected subset and the Spearman correlation was computed between the adjusted cytokine values of this subset, and their corresponding adjusted values over the entire set of 37 cytokines. The graph presents the average median correlation across all 50 subsets, where the median was computed for each subset across all cytokines tested.

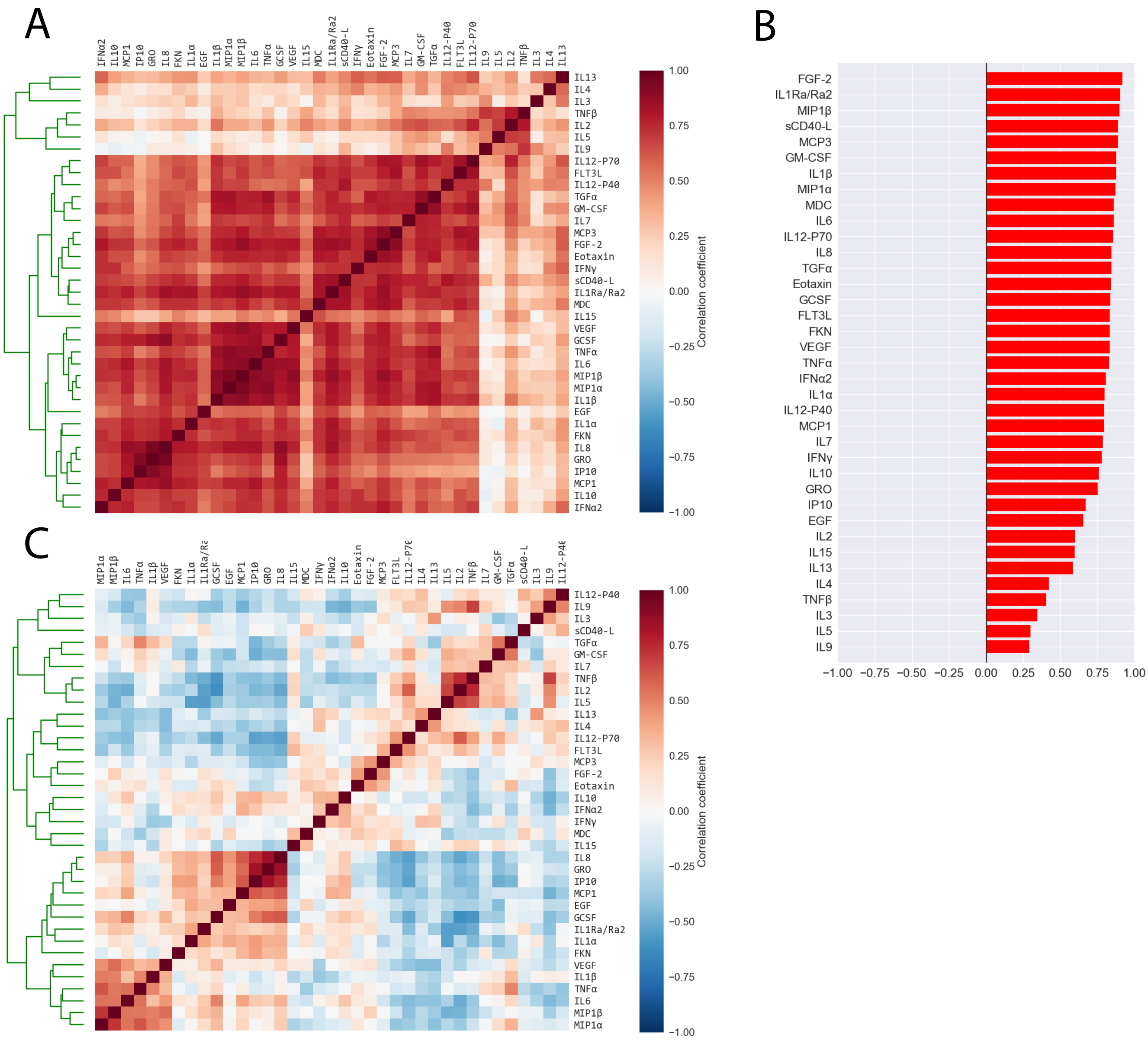

Figure S2. Cytokine levels are highly correlated to each other and to the mean cytokine level of each subject. (A) Pairwise Pearson's correlations among the absolute nasal wash cytokine levels in the FLU09 cohort. Cytokines were sorted along both axes using hierarchical clustering (complete-linkage). (B) Correlations between cytokine levels and mean cytokine levels for each subject. (C) Pairwise Pearson's correlations between cytokines following adjustment to the mean cytokine level (see Methods for details). Cytokines were sorted along both axes using complete-linkage.

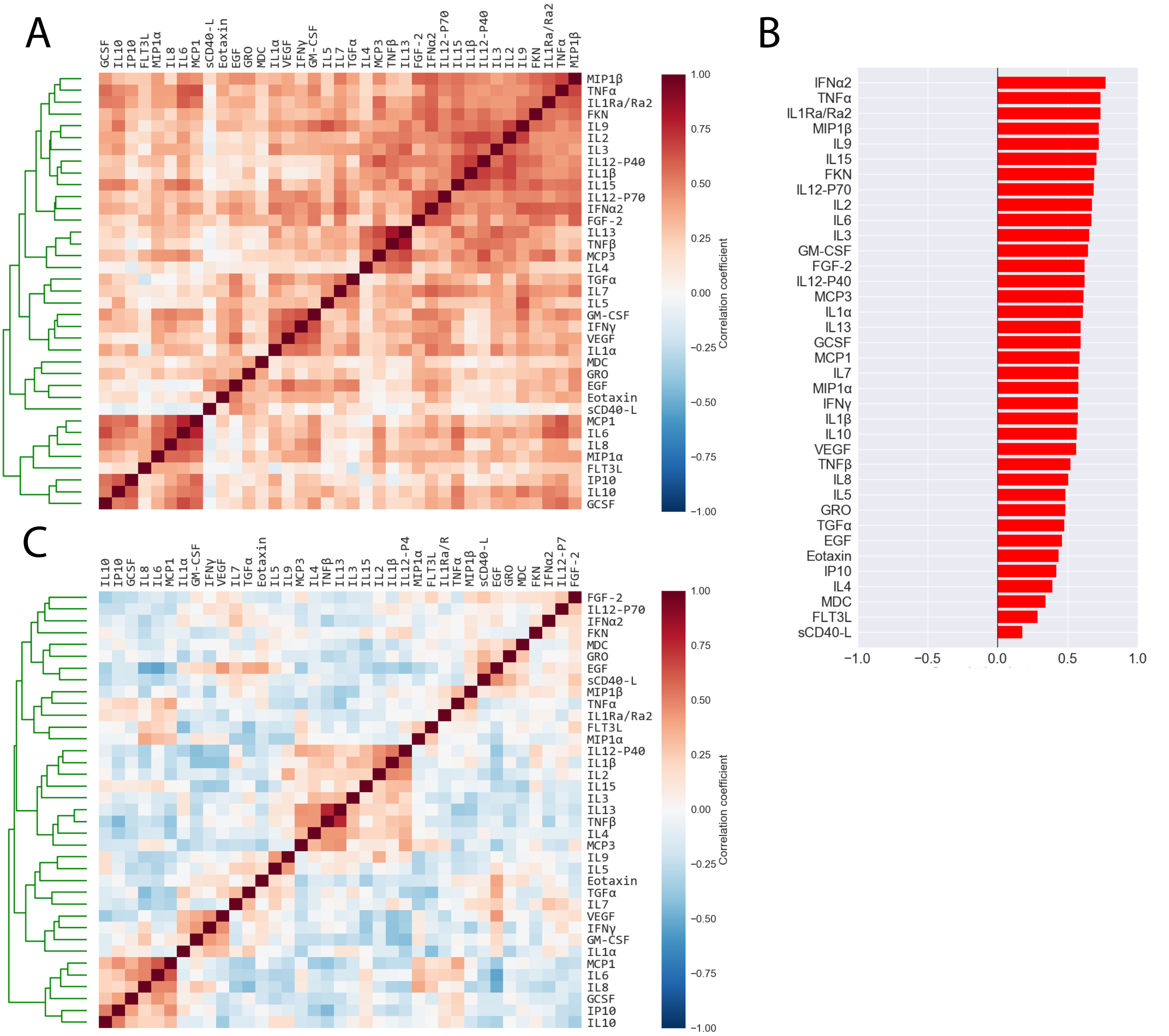

Figure S3. Cytokine levels are highly correlated to each other and to the mean cytokine level of each subject. (A) Pairwise Pearson's correlations among the absolute serum cytokine levels in the PICFLU cohort. Cytokines were sorted along both axes using hierarchical clustering (complete-linkage). (B) Correlations between cytokine levels and mean cytokine levels for each subject. (C) Pairwise Pearson's correlations between cytokines following adjustment to the mean cytokine level (see Methods for details). Cytokines were sorted along both axes using complete-linkage.

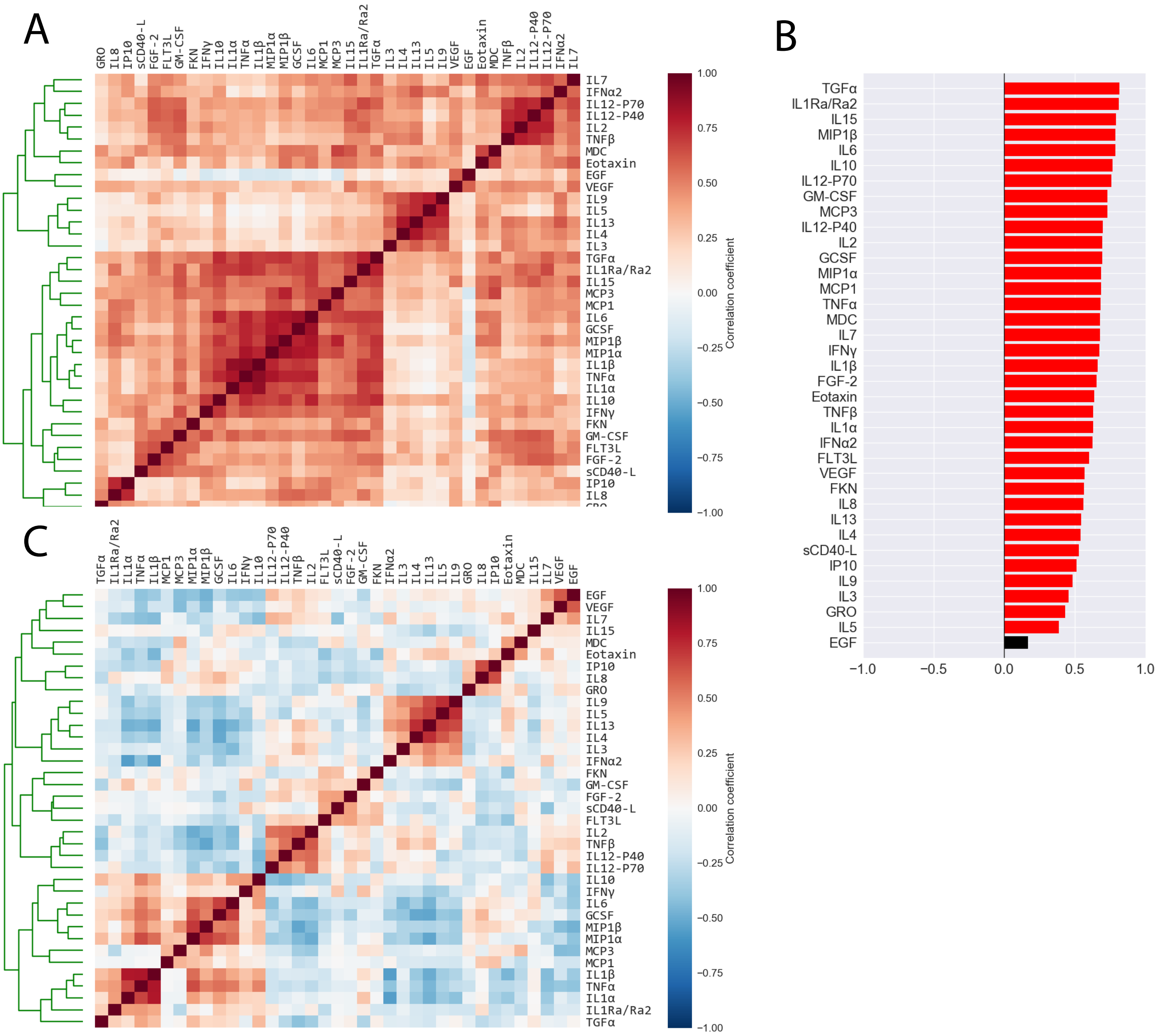

Figure S4. Cytokine levels are highly correlated to each other and to the mean cytokine level of each subject. (A) Pairwise Pearson's correlations among the absolute endotracheal cytokine levels in the PICFLU cohort. Cytokines were sorted along both axes using hierarchical clustering (complete-linkage). (B) Correlations between cytokine levels and mean cytokine levels for each subject. (C) Pairwise Pearson's correlations between cytokines following adjustment to the mean cytokine level (see Methods for details). Cytokines were sorted along both axes using complete-linkage.

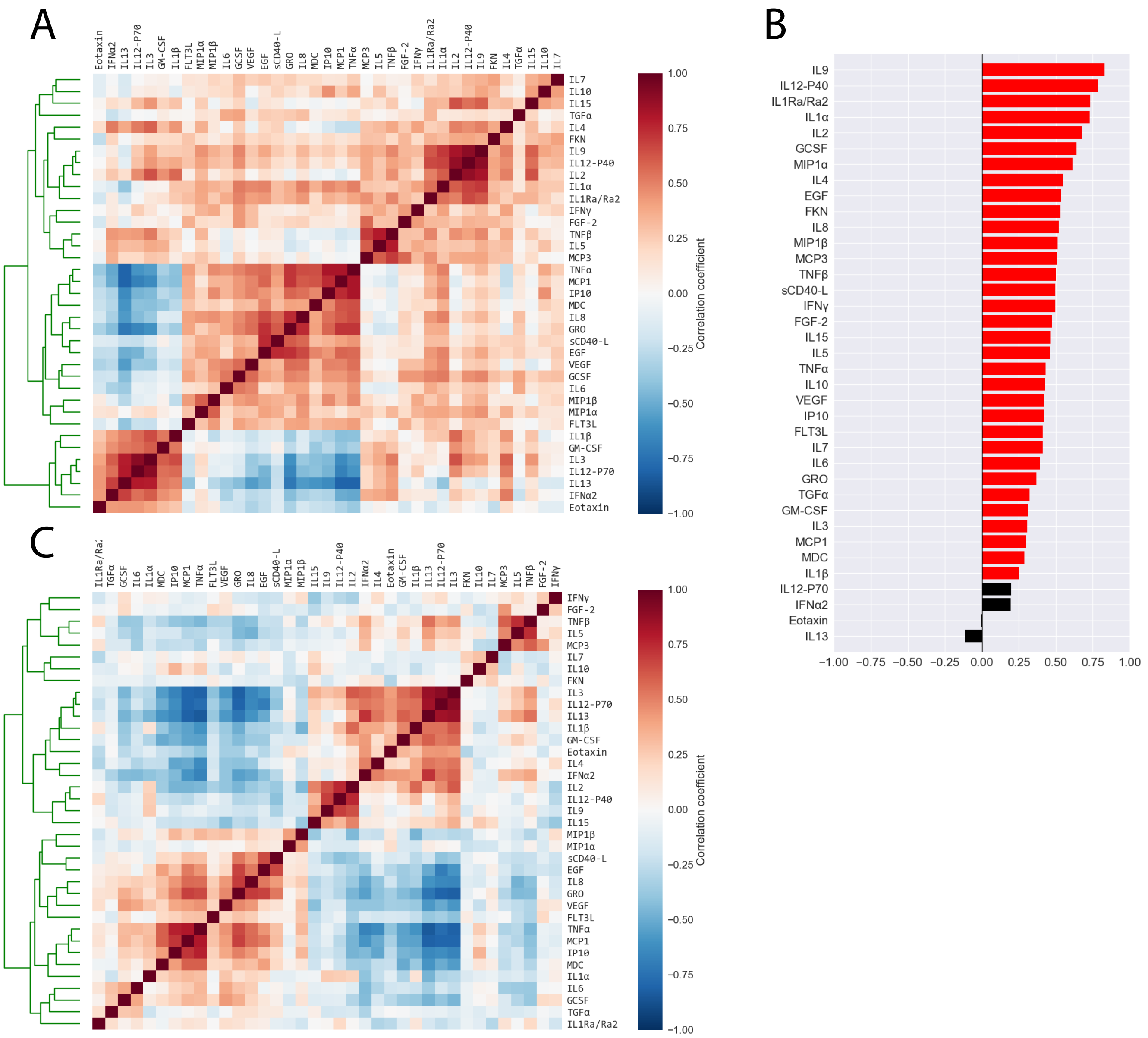

Figure S5. Cytokine levels are highly correlated to each other and to the mean cytokine level of each subject. (A) Pairwise Pearson's correlations among the absolute serum cytokine levels in the SHIVERS cohort. Cytokines were sorted along both axes using hierarchical clustering (complete-linkage). (B) Correlations between cytokine levels and mean cytokine levels for each subject. (C) Pairwise Pearson's correlations between cytokines following adjustment to the mean cytokine level (see Methods for details). Cytokines were sorted along both axes using complete-linkage.

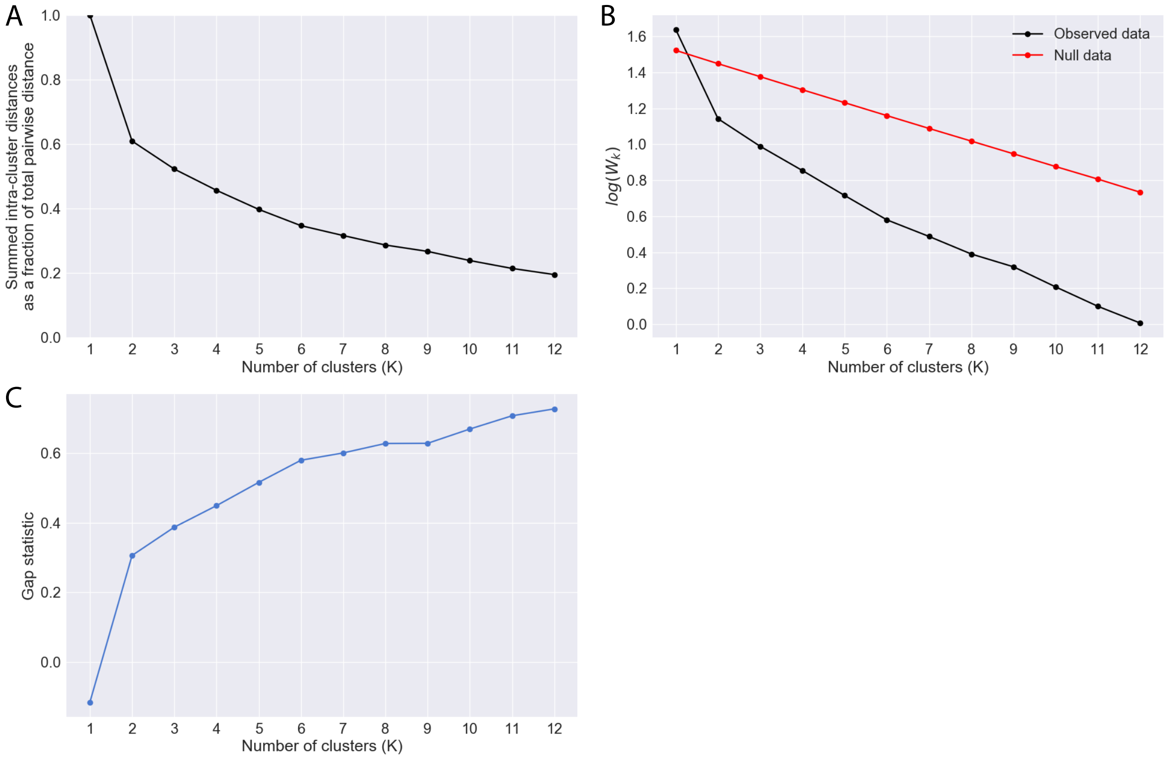

Figure S6. Computing the Tibshirani Gap statistic for the FLU09 adjusted plasma cytokine profiles. (A) Summed intra-cluster distances as a fraction of total pairwise distances for each possible K between 1-12. (B) The log of pooled sum of intra-cluster distances (W_k) for each possible K between 1-12. (C) Gap statistic value for each possible K between 1-12.

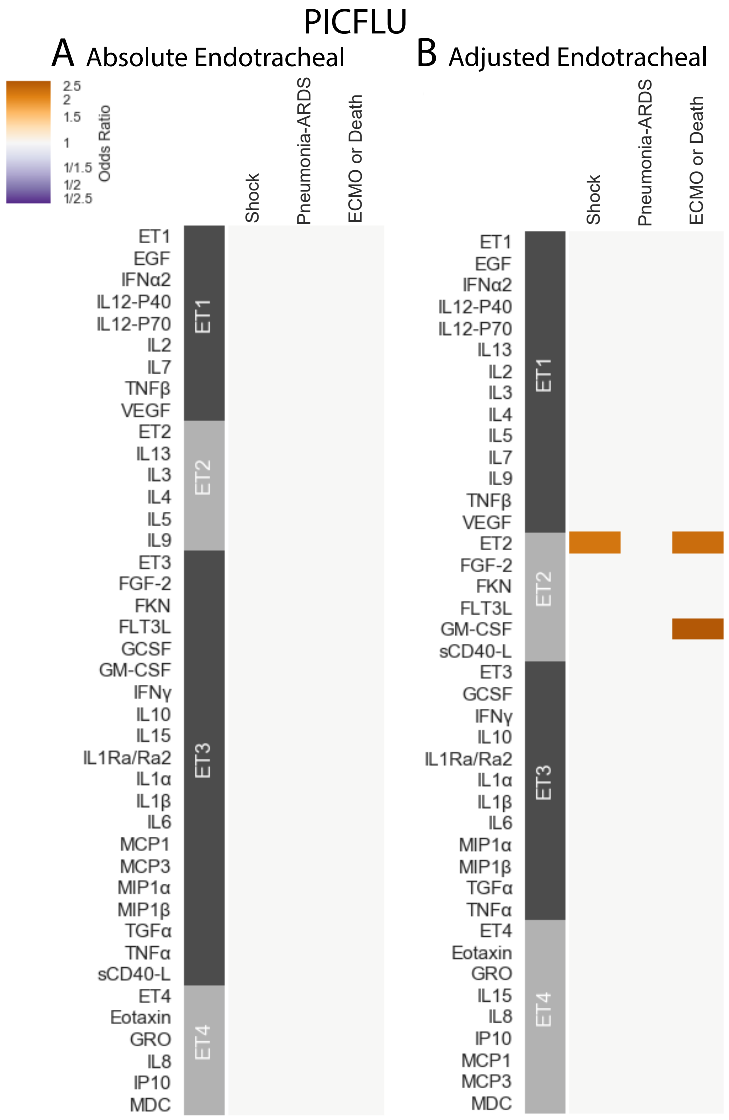

Figure S7. PICFLU endotracheal cytokine associations with clinical phenotypes identified using logistic regression while also controlling for patients age and bacterial coinfection. Modules constructed of covarying cytokines (absolute and adjusted measurements separately) from serum samples, were tested for associations with the clinical phenotypes described in Section 2.1: shock, pneumonia-ARDS and ECMO or death. Each cytokine or module is indicated along the rows, grouped by their assigned module. Heatmap color indicates the direction and magnitude of the regression coefficient between cytokine or module level with a given clinical phenotype. Only associations with false-discovery rate (FDR)-adjusted q-value $\leq$ 0.2 are colored. Asterisks indicate family-wise error rate (FWER)-adjusted p-values with ***, **, and * indicating p $\leq$ 0.0005, 0.005, and 0.05, respectively.

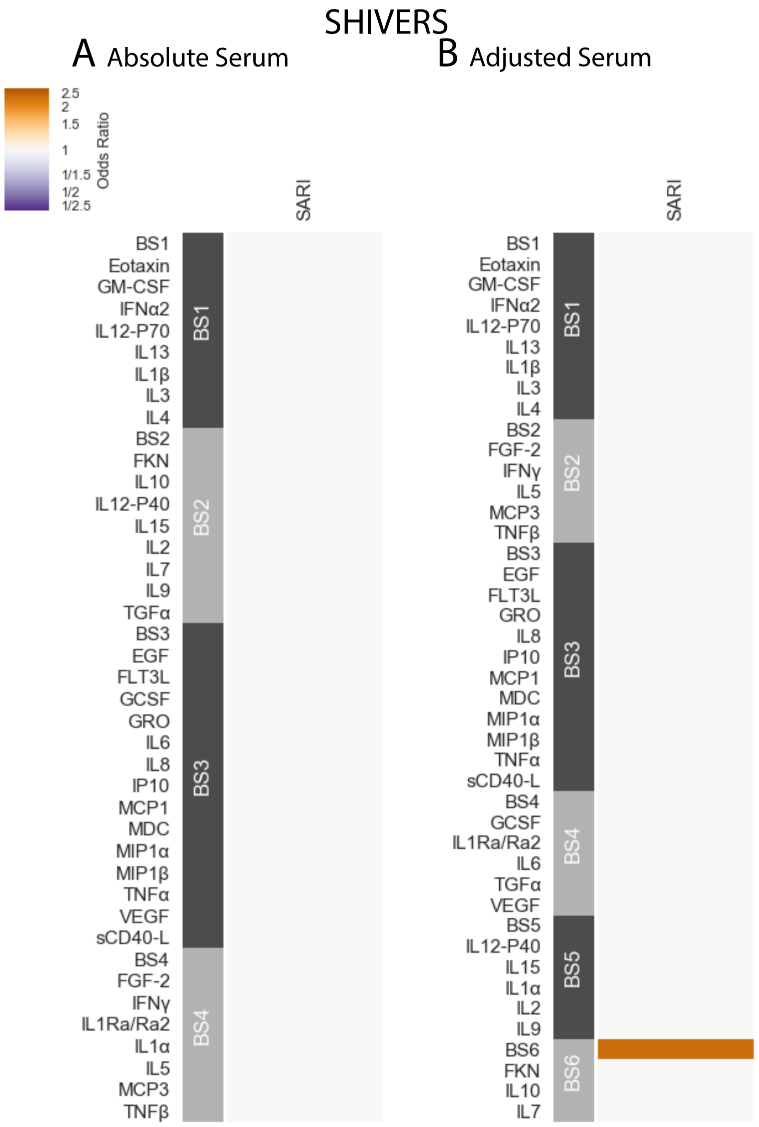

Figure S8. SHIVERS serum cytokine associations with phenotypes estimated using linear regression while also controlling for patients age, ethnicity and sampling time. Modules constructed of covarying cytokines (absolute and adjusted measurements separately) from serum samples, were tested for associations with the clinical phenotypes described in Section 2.1. Each cytokine or module is indicated along the rows, grouped by their assigned module. Heatmap color indicates the direction and magnitude of the regression coefficient between cytokine or module level with a given clinical phenotype. Only associations with false-discovery rate (FDR)-adjusted q-value ≤ 0.2 are colored. Asterisks indicate family-wise error rate (FWER)-adjusted p-values with ***, **, and * indicating $p \leq 0.0005$, 0.005, and 0.05, respectively.

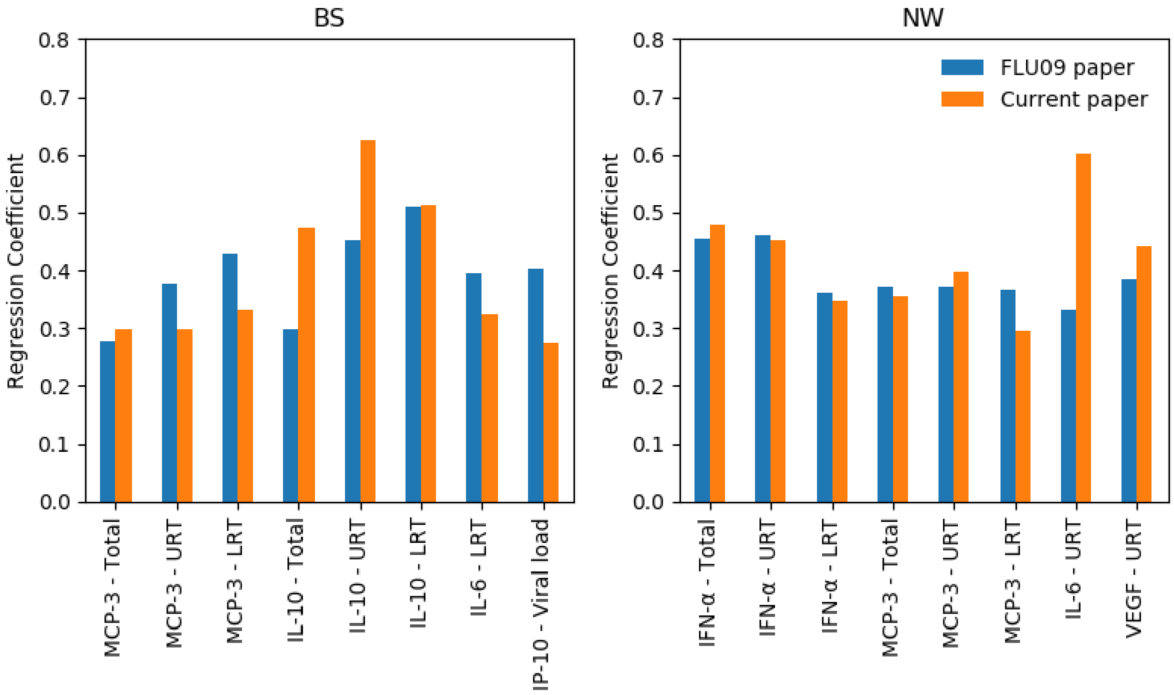

Figure S9. A comparison of the regression coefficients of associations of single cytokine measurements with clinical symptom scores that were previously reported to be significantly associated (FDR q-value $\leq$ 0.2) in the FLU09 dataset assessed using data from years 1-2 (Oshansky et al., 2014) and the complete dataset (years 1-5). The FDR q-values for each cytokine over the full dataset were lower than 0.2.

1.2 Tables

Table S1: FLU09 absolute plasma modules and individual cytokine associations with phenotypes

Outcome	Module/Analyte	Coef	P-value	FWER	FDR
Total	BS3	0.529	0.000147	**0.00354**	0.00191
Systemic	BS3	0.605	0.000159	**0.00366**	0.00191
Lower RT	BS4	0.322	0.00138	**0.0304**	0.0111
Total	BS4	0.355	0.00482	0.101	0.0289
Upper RT	BS4	0.342	0.0092	0.184	0.0367
Upper RT	BS3	0.394	0.0102	0.194	0.0367
Lower RT	BS2	0.281	0.0107	0.194	0.0367
Systemic	BS4	0.334	0.0244	0.414	0.0731
Upper RT	BS2	0.3	0.0348	0.557	0.0929
Total	GRO	0.556	2.23e-05	**0.00495**	0.00279
Systemic	IP10	0.635	2.99e-05	**0.0066**	0.00279
Lower RT	IL10	0.513	3.78e-05	**0.00831**	0.00279
Total	IP10	0.534	6.63e-05	**0.0145**	0.00368
Upper RT	IL10	0.625	9.39e-05	**0.0205**	0.00375
Lower RT	FKN	0.369	0.000101	**0.022**	0.00375
Total	EGF	0.524	0.000171	**0.037**	0.00544
Lower RT	MIP1β	0.353	0.000383	0.0824	0.0106
Upper RT	GRO	0.497	0.000432	0.0925	0.0107
Systemic	EGF	0.552	0.000753	0.16	0.0167
Lower RT	MCP3	0.331	0.00103	0.218	0.0208
Lower RT	IL1α	0.369	0.00123	0.259	0.0227
Systemic	GRO	0.51	0.00142	0.299	0.0243
Lower RT	IL6	0.324	0.00178	0.373	0.0283
Upper RT	TNFα	0.384	0.0022	0.458	0.0326
Lower RT	GRO	0.346	0.00246	0.509	0.0334
Systemic	TGFα	0.444	0.00255	0.526	0.0334
Upper RT	MIP1β	0.386	0.00294	0.603	0.0354
log-VL	IP10	0.275	0.00303	0.618	0.0354
Total	IL1α	0.414	0.00367	0.746	0.0391
Total	IL10	0.474	0.0037	0.748	0.0391
Total	IL6	0.368	0.00435	0.875	0.0422
Systemic	MCP1	0.47	0.00437	0.875	0.0422
Lower RT	GM-CSF	0.294	0.00562	1	0.052
Upper RT	FKN	0.351	0.00614	1	0.0542
Total	MIP1β	0.347	0.00634	1	0.0542
Lower RT	TNFα	0.272	0.00664	1	0.0546
Total	TNFα	0.329	0.00772	1	0.0612
Systemic	IL8	0.344	0.00818	1	0.0626
Systemic	Eotaxin	0.372	0.00856	1	0.0634
Total	IL8	0.297	0.00889	1	0.0636
Upper RT	GCSF	0.409	0.00944	1	0.0639
Total	FKN	0.323	0.0095	1	0.0639
Systemic	IL6	0.386	0.0102	1	0.0666
Lower RT	EGF	0.304	0.0119	1	0.0754
Upper RT	IP10	0.371	0.0132	1	0.0814
Upper RT	IL6	0.334	0.014	1	0.0841
Lower RT	FGF-2	0.246	0.0156	1	0.0897
Upper RT	EGF	0.372	0.0158	1	0.0897

Continued on next page

Table S1: FLU09 absolute plasma modules and individual cytokine associations with phenotypes

Outcome	Module/Analyte	Coef	P-value	FWER	FDR
Lower RT	GCSF	0.299	0.0172	1	0.0952
Total	IFNα2	0.317	0.0197	1	0.104
Upper RT	IL1α	0.35	0.0206	1	0.104
Total	MCP1	0.34	0.0208	1	0.104
Lower RT	IL12-P70	0.239	0.0211	1	0.104
Total	TGFα	0.304	0.0214	1	0.104
Total	MCP3	0.298	0.0219	1	0.104
Upper RT	IFNα2	0.322	0.022	1	0.104
Total	Eotaxin	0.279	0.0255	1	0.118
Upper RT	MCP3	0.298	0.0267	1	0.121
Systemic	IL1α	0.367	0.0302	1	0.134
log-VL	GRO	0.204	0.0327	1	0.14
Lower RT	IFNα2	0.238	0.0328	1	0.14
Lower RT	IP10	0.253	0.0339	1	0.142
Total	sCD40-L	0.312	0.0379	1	0.154
Total	GCSF	0.323	0.038	1	0.154
Upper RT	IL8	0.243	0.0428	1	0.17

Table S2: FLU09 adjusted plasma modules and individual cytokine associations with phenotypes

Outcome	Module/Analyte	Coef	P-value	FWER	FDR
Total	BS1	-0.557	3.15e-05	**0.00114**	0.00114
Systemic	BS1	-0.582	0.000229	**0.00803**	0.00413
Lower RT	BS1	-0.35	0.00252	0.0858	0.0281
Systemic	BS3	0.461	0.00312	0.103	0.0281
Upper RT	BS1	-0.42	0.00468	0.15	0.0337
Total	BS3	0.348	0.0121	0.376	0.0729
Lower RT	BS4	0.252	0.0146	0.439	0.0752
Systemic	IL9	-0.634	0.000428	0.0949	0.0491
Lower RT	IL4	-0.319	0.000847	0.187	0.0491
Systemic	IP10	0.512	0.000889	0.196	0.0491
Total	IL7	-0.449	0.00111	0.242	0.0491
Total	IL9	-0.512	0.00131	0.286	0.0491
Total	IL4	-0.377	0.00145	0.314	0.0491
Upper RT	IL4	-0.387	0.00159	0.344	0.0491
Total	IL1β	-0.526	0.00177	0.381	0.0491
Systemic	IL7	-0.495	0.0021	0.45	0.0519
Total	GRO	0.425	0.00235	0.501	0.0522
Lower RT	FKN	0.282	0.00286	0.606	0.0577
Systemic	IL5	-0.459	0.0032	0.675	0.0592
Total	IP10	0.399	0.00357	0.75	0.0606
Systemic	EGF	0.556	0.00382	0.798	0.0606
Systemic	IL1β	-0.562	0.00411	0.855	0.0608
Total	EGF	0.475	0.00466	0.965	0.0646
Gastrointestinal	EGF	0.51	0.00498	1	0.0651

Continued on next page

Table S2: FLU09 adjusted plasma modules and individual cytokine associations with phenotypes

Outcome	Module/Analyte	Coef	P-value	FWER	FDR
Total	IL5	-0.378	0.00569	1	0.0693
log-VL	IP10	0.25	0.00593	1	0.0693
Systemic	TNFβ	-0.421	0.00777	1	0.0828
Total	TNFβ	-0.366	0.00784	1	0.0828
Lower RT	IL7	-0.303	0.00879	1	0.0887
Lower RT	IL10	0.357	0.0141	1	0.133
Upper RT	IL10	0.449	0.015	1	0.133
Systemic	TGFα	0.375	0.0153	1	0.133
Lower RT	MIP1β	0.257	0.0155	1	0.133
Gastrointestinal	GRO	0.366	0.0167	1	0.135
Total	IL13	-0.369	0.0183	1	0.135
Systemic	GRO	0.391	0.0184	1	0.135
Upper RT	GRO	0.35	0.0188	1	0.135
Lower RT	IL9	-0.317	0.0189	1	0.135
Lower RT	MCP3	0.257	0.0212	1	0.147
Lower RT	IL1β	-0.32	0.0247	1	0.166
Systemic	IL13	-0.394	0.0295	1	0.192
Systemic	IL4	-0.309	0.0303	1	0.192
Systemic	MCP1	0.344	0.0324	1	0.2
Systemic	Eotaxin	0.309	0.0338	1	0.203
log-VL	IL12-P40	-0.199	0.0371	1	0.217
Upper RT	IL1β	-0.368	0.0441	1	0.245
Systemic	IL8	0.264	0.0441	1	0.245
Upper RT	IL7	-0.298	0.0479	1	0.259

Table S3: FLU09 absolute nasal wash modules and individual cytokine associations with phenotypes

Outcome	Module/Analyte	Coef	P-value	FWER	FDR
Upper RT	NW2	0.46	0.00162	**0.0292**	0.0292
log-VL	NW3	0.202	0.00486	0.0827	0.0438
Total	NW2	0.394	0.00844	0.135	0.0507
Lower RT	NW2	0.266	0.0479	0.718	0.173
Gastrointestinal	NW2	0.336	0.0479	0.718	0.173
Upper RT	IL6	0.601	2.36e-05	**0.00523**	0.00523
Upper RT	IL10	0.436	0.00026	0.0575	0.0244
Total	IFNα2	0.479	0.00033	0.0727	0.0244
Total	IL6	0.517	0.000609	0.133	0.0279
Upper RT	IFNα2	0.453	0.000629	0.137	0.0279
Upper RT	MIP1β	0.51	0.00126	0.274	0.0418
Total	IL10	0.39	0.00148	0.319	0.0418
Upper RT	VEGF	0.441	0.00151	0.324	0.0418
log-VL	TNFβ	0.216	0.00247	0.529	0.061
Upper RT	GCSF	0.447	0.00281	0.598	0.0623
Lower RT	IL6	0.388	0.00356	0.755	0.0719
Upper RT	IL1α	0.365	0.00433	0.914	0.0734

Continued on next page

Table S3: FLU09 absolute nasal wash modules and individual cytokine associations with phenotypes

Outcome	Module/Analyte	Coef	P-value	FWER	FDR
Lower RT	IL10	0.311	0.00449	0.944	0.0734
Total	FLT3L	0.342	0.00463	0.967	0.0734
Lower RT	IFNα2	0.349	0.00568	1	0.0802
Upper RT	FGF-2	0.433	0.00591	1	0.0802
Upper RT	FLT3L	0.329	0.00614	1	0.0802
Total	IL1α	0.35	0.00693	1	0.0854
Upper RT	MCP3	0.397	0.00731	1	0.0854
log-VL	IL5	0.186	0.00843	1	0.0914
Total	IFNγ	0.331	0.00896	1	0.0914
Upper RT	IFNγ	0.327	0.00905	1	0.0914
Upper RT	Eotaxin	0.354	0.00963	1	0.093
Lower RT	MIP1β	0.37	0.0101	1	0.0933
Upper RT	FKN	0.317	0.0119	1	0.105
Upper RT	IL1β	0.342	0.0132	1	0.105
Upper RT	MDC	0.322	0.0134	1	0.105
Lower RT	GCSF	0.333	0.0138	1	0.105
Systemic	IFNα2	0.367	0.0145	1	0.105
Total	MDC	0.321	0.0145	1	0.105
Total	MIP1β	0.4	0.0146	1	0.105
Systemic	FLT3L	0.313	0.0154	1	0.107
log-VL	IL2	0.179	0.0165	1	0.111
Total	MCP3	0.355	0.0185	1	0.121
Systemic	IFNγ	0.315	0.0193	1	0.123
Gastrointestinal	MDC	0.329	0.023	1	0.129
Systemic	IL10	0.303	0.0232	1	0.129
Total	GCSF	0.35	0.0238	1	0.129
Upper RT	TNFα	0.346	0.0239	1	0.129
Gastrointestinal	Eotaxin	0.341	0.0246	1	0.129
log-VL	EGF	-0.171	0.0269	1	0.129
Lower RT	MCP3	0.295	0.0272	1	0.129
Upper RT	IL12-P40	0.259	0.0275	1	0.129
Upper RT	MCP1	0.31	0.0275	1	0.129
Total	FGF-2	0.356	0.0276	1	0.129
Upper RT	IL8	0.321	0.0277	1	0.129
Gastrointestinal	IFNα2	0.342	0.0283	1	0.129
Gastrointestinal	IL12-P40	0.291	0.0283	1	0.129
log-VL	TNFα	0.196	0.0285	1	0.129
Total	FKN	0.279	0.0294	1	0.131
Systemic	IL15	0.267	0.0303	1	0.132
Lower RT	IL1α	0.251	0.0311	1	0.133
Total	IL15	0.248	0.033	1	0.136
Total	IL12-P40	0.254	0.0331	1	0.136
log-VL	IL6	0.191	0.0373	1	0.15
Total	IP10	0.284	0.0378	1	0.15
log-VL	GM-CSF	0.179	0.0386	1	0.15
Gastrointestinal	IL6	0.361	0.0396	1	0.152
Upper RT	IL12-P70	0.249	0.0427	1	0.16
Total	VEGF	0.293	0.0432	1	0.16
Total	Eotaxin	0.28	0.0455	1	0.162

Continued on next page

Table S3: FLU09 absolute nasal wash modules and individual cytokine associations with phenotypes

Outcome	Module/Analyte	Coef	P-value	FWER	FDR
log-VL	IFNα2	0.176	0.0458	1	0.162
Total	MCP1	0.288	0.0459	1	0.162
Upper RT	IP10	0.269	0.0472	1	0.162
Upper RT	IL1Ra/Ra2	0.299	0.0474	1	0.162
Systemic	FKN	0.269	0.048	1	0.162

Table S4: FLU09 adjusted nasal wash modules and individual cytokine associations with phenotypes

Outcome	Module/Analyte	Coef	P-value	FWER	FDR
log-VL	NW6	-0.211	0.00393	0.141	0.141
Systemic	NW2	0.282	0.0107	0.375	0.193
Total	NW2	0.247	0.0189	0.642	0.226
Upper RT	NW2	0.206	0.0499	1	0.449
log-VL	EGF	-0.281	7.07e-05	**0.0157**	0.0157
log-VL	IL1Ra/Ra2	-0.215	0.00313	0.692	0.248
log-VL	Eotaxin	-0.21	0.00335	0.737	0.248
Systemic	FLT3L	0.342	0.00481	1	0.267
log-VL	TNFβ	0.194	0.00651	1	0.289
log-VL	MDC	-0.184	0.00988	1	0.365
log-VL	FGF-2	-0.18	0.0155	1	0.402
log-VL	IL5	0.171	0.0169	1	0.402
Lower RT	GM-CSF	-0.241	0.0172	1	0.402
Upper RT	IL6	0.27	0.0195	1	0.402
Upper RT	IL10	0.249	0.0199	1	0.402
Lower RT	IL7	-0.243	0.0251	1	0.464
Total	IL10	0.231	0.033	1	0.472
Total	FLT3L	0.244	0.0372	1	0.472
Total	IFNα2	0.209	0.039	1	0.472
Systemic	TGFα	-0.266	0.0401	1	0.472
Lower RT	IL10	0.197	0.0403	1	0.472
Systemic	IFNγ	0.24	0.0419	1	0.472
Total	IL5	-0.246	0.0426	1	0.472
log-VL	IL1α	-0.147	0.0448	1	0.472
Upper RT	IL4	-0.221	0.0458	1	0.472
Systemic	IL10	0.229	0.0468	1	0.472

Table S5: PICFLU absolute serum modules and individual cytokine associations with phenotypes

Outcome	Module/Analyte	Odds Ratio	P-value	FWER	FDR
Shock	BS3	2.75	1.62e-06	**2.43e-05**	2.43e-05
ECMO or Death	BS3	2.04	0.00204	**0.0286**	0.0153
Pneumonia-ARDS	BS3	1.61	0.00693	0.0901	0.0347

Continued on next page

Table S5: PICFLU absolute serum modules and individual cytokine associations with phenotypes

Outcome	Module/Analyte	Odds Ratio	P-value	FWER	FDR
ECMO or Death	BS4	0.643	0.0286	0.343	0.107
Shock	MCP1	2.62	4.35e-07	**4.83e-05**	4.83e-05
Shock	IL6	2.12	5.18e-05	**0.0057**	0.00288
Shock	IL8	2.37	8.84e-05	**0.00964**	0.00314
Shock	IP10	1.95	0.000113	**0.0122**	0.00314
Pneumonia-ARDS	MCP1	1.85	0.000283	**0.0303**	0.00625
Pneumonia-ARDS	IL8	2.17	0.000338	**0.0358**	0.00625
ECMO or Death	MIP1α	1.97	0.000766	0.0804	0.0113
ECMO or Death	IL8	2.95	0.000818	0.0851	0.0113
ECMO or Death	IL6	2.45	0.00128	0.132	0.0158
Shock	IL10	1.69	0.0015	0.153	0.0167
Shock	TNFα	1.66	0.0026	0.263	0.0256
ECMO or Death	MCP1	1.98	0.00276	0.276	0.0256
Shock	GCSF	1.61	0.00489	0.484	0.0418
ECMO or Death	EGF	0.437	0.00608	0.596	0.0479
ECMO or Death	MDC	0.545	0.00683	0.662	0.0479
ECMO or Death	sCD40-L	0.549	0.00691	0.663	0.0479
ECMO or Death	IL10	1.75	0.0113	1	0.0738
Pneumonia-ARDS	FGF-2	0.65	0.0141	1	0.0872
ECMO or Death	FKN	1.69	0.0179	1	0.105
Shock	MIP1α	1.43	0.02	1	0.111
Pneumonia-ARDS	IL10	1.44	0.0218	1	0.112
ECMO or Death	IL1β	1.61	0.0225	1	0.112
Pneumonia-ARDS	IL6	1.47	0.0232	1	0.112
ECMO or Death	IL12-P40	1.6	0.03	1	0.139
Pneumonia-ARDS	MDC	0.709	0.0354	1	0.157
Shock	EGF	0.694	0.037	1	0.158
ECMO or Death	IL15	1.8	0.0416	1	0.161
ECMO or Death	IL1Ra/Ra2	1.6	0.044	1	0.161
Shock	IL12-P70	0.735	0.0452	1	0.161
Pneumonia-ARDS	MIP1α	1.36	0.0461	1	0.161
Shock	sCD40-L	0.745	0.0463	1	0.161
ECMO or Death	TNFα	1.6	0.0463	1	0.161

Table S6: PICFLU adjusted serum modules and individual cytokine associations with phenotypes

Outcome	Module/Analyte	Odds Ratio	P-value	FWER	FDR
Shock	BS3	3.01	1.73e-07	**2.08e-06**	2.08e-06
Shock	BS4	0.463	1.97e-05	**0.000217**	0.000118
ECMO or Death	BS3	2.46	0.000426	**0.00426**	0.00171
Pneumonia-ARDS	BS3	1.75	0.00106	**0.00957**	0.00319
Pneumonia-ARDS	BS4	0.598	0.00194	**0.0155**	0.00465
ECMO or Death	BS4	0.494	0.00404	**0.0283**	0.00808
ECMO or Death	BS2	0.586	0.0123	0.0738	0.0211
Shock	MCP1	2.7	1.31e-07	**1.45e-05**	1.45e-05

Continued on next page

Table S6: PICFLU adjusted serum modules and individual cytokine associations with phenotypes

Outcome	Module/Analyte	Odds Ratio	P-value	FWER	FDR
Shock	IL6	2.18	1.27e-05	**0.00139**	0.000703
Pneumonia-ARDS	MCP1	1.95	6.1e-05	**0.00665**	0.00226
Shock	IL8	2.16	0.000136	**0.0147**	0.00368
Pneumonia-ARDS	IL8	2.21	0.000166	**0.0177**	0.00368
Shock	IP10	1.86	0.00022	**0.0234**	0.00408
ECMO or Death	EGF	0.211	0.000409	**0.043**	0.00649
Shock	IL12-P70	0.565	0.000491	0.0511	0.00682
ECMO or Death	IL8	3.41	0.000623	0.0641	0.00768
ECMO or Death	MIP1α	1.99	0.000818	0.0834	0.00908
ECMO or Death	IL6	2.52	0.00112	0.113	0.0113
Shock	TNFα	1.7	0.00122	0.122	0.0113
ECMO or Death	MDC	0.443	0.00187	0.185	0.0151
Shock	IL10	1.6	0.00191	0.187	0.0151
ECMO or Death	sCD40-L	0.511	0.00271	0.263	0.02
ECMO or Death	MCP1	2.06	0.00423	0.406	0.0293
Shock	IL7	0.65	0.00452	0.429	0.0295
Pneumonia-ARDS	FGF-2	0.598	0.00499	0.469	0.0308
ECMO or Death	IFNγ	0.512	0.00603	0.561	0.0352
Pneumonia-ARDS	IL6	1.53	0.00859	0.79	0.0449
Shock	EGF	0.616	0.00893	0.813	0.0449
ECMO or Death	VEGF	0.502	0.00906	0.815	0.0449
Shock	GCSF	1.52	0.0093	0.828	0.0449
Pneumonia-ARDS	IL10	1.45	0.0136	1	0.062
Shock	FGF-2	0.646	0.014	1	0.062
Pneumonia-ARDS	IL2	0.688	0.0166	1	0.0707
Pneumonia-ARDS	MDC	0.664	0.0185	1	0.0761
ECMO or Death	IL10	1.72	0.0216	1	0.0856
Shock	GRO	0.713	0.0229	1	0.0877
Shock	IFNα2	0.714	0.0271	1	0.0983
Shock	sCD40-L	0.718	0.0274	1	0.0983
Pneumonia-ARDS	IFNα2	0.717	0.0305	1	0.105
ECMO or Death	IL15	1.97	0.0313	1	0.105
Shock	TGFα	0.694	0.0345	1	0.111
Pneumonia-ARDS	MIP1α	1.37	0.0351	1	0.111
ECMO or Death	FKN	1.59	0.0359	1	0.111
Shock	MIP1α	1.37	0.0378	1	0.113
ECMO or Death	IL1β	1.55	0.0485	1	0.142

Table S7: PICFLU absolute endotracheal aspirate modules and individual cytokine associations with phenotypes

Outcome	Module/Analyte	Odds Ratio	P-value	FWER	FDR
Shock	FLT3L	2.52	0.00328	0.364	0.26
Shock	FGF-2	2.4	0.00469	0.516	0.26
ECMO or Death	GM-CSF	2.78	0.0075	0.818	0.272
Shock	sCD40-L	2.12	0.0112	1	0.272
ECMO or Death	EGF	0.418	0.0123	1	0.272

Continued on next page

Table S7: PICFLU absolute endotracheal aspirate modules and individual cytokine associations with phenotypes

Outcome	Module/Analyte	Odds Ratio	P-value	FWER	FDR
Shock	GM-CSF	2.46	0.0168	1	0.31
Shock	IL12-P40	1.8	0.0283	1	0.449
Pneumonia-ARDS	TNFα	0.583	0.0428	1	0.488

Table S8: PICFLU adjusted endotracheal aspirate modules and indiviudal cytokine associations with phenotypes

Outcome	Module/Analyte	Odds Ratio	P-value	FWER	FDR
Shock	ET2	2.27	0.00596	0.0715	0.05
ECMO or Death	ET2	2.38	0.00833	0.0916	0.05
ECMO or Death	GM-CSF	3.02	0.0015	0.166	0.166
ECMO or Death	EGF	0.386	0.00849	0.934	0.315
Shock	FLT3L	2.15	0.0108	1	0.315
Shock	FGF-2	2.01	0.0113	1	0.315
Pneumonia-ARDS	TGFα	0.484	0.0146	1	0.325
Shock	IL5	0.545	0.0303	1	0.358
Shock	IL10	0.578	0.0313	1	0.358
Pneumonia-ARDS	TNFα	0.572	0.0317	1	0.358
ECMO or Death	IL1Ra/Ra2	0.426	0.033	1	0.358
Shock	sCD40-L	1.82	0.0381	1	0.358

Table S9: SHIVERS absolute serum modules and individual cytokine associations with phenotypes

Outcome	Module/Analyte	Odds Ratio	P-value	FWER	FDR
SARI	MDC	0.367	0.0288	1	0.707

Table S10: SHIVERS adjusted serum modules and individual cytokine associations with phenotypes

Outcome	Module/Analyte	Odds Ratio	P-value	FWER	FDR
SARI	BS6	2.4	0.028	0.168	0.168
SARI	GRO	2.96	0.0249	0.921	0.337
SARI	FKN	2.47	0.0312	1	0.337
SARI	IL12-P40	0.402	0.0345	1	0.337
SARI	MDC	0.413	0.0364	1	0.337
SARI	VEGF	5.67	0.0476	1	0.348

Table S11: FLU09 absolute plasma cores and individual cytokine associations with phenotypes

Outcome	Core	Coef	P-value	FWER	FDR
Systemic	Core5	0.6	0.000131	**0.0055**	0.0055
Total	Core5	0.475	0.000685	**0.0281**	0.0102
Lower RT	Core4	0.328	0.000836	**0.0334**	0.0102
Total	Core1	0.522	0.00116	**0.045**2	0.0102
Upper RT	Core4	0.406	0.00121	**0.046**	0.0102
Total	Core6	0.368	0.00378	0.14	0.0235
Total	Core4	0.356	0.00392	0.141	0.0235
Systemic	Core1	0.532	0.00475	0.166	0.0249
Systemic	Core6	0.393	0.00775	0.263	0.0362
Upper RT	Core6	0.346	0.00934	0.308	0.0392
Lower RT	Core6	0.266	0.0115	0.369	0.044
Upper RT	Core1	0.416	0.016	0.495	0.0559
Upper RT	Core5	0.335	0.0285	0.856	0.0921
log-VL	Core5	0.197	0.0395	1	0.119

Table S12: FLU09 adjusted plasma cores and individual cytokine associations with phenotypes

Outcome	Core	Coef	P-value	FWER	FDR
Total	Core3	-0.412	0.00262	0.0944	0.0944
Systemic	Core3	-0.409	0.0112	0.391	0.142
Gastrointestinal	Core1	0.386	0.0168	0.57	0.142
Systemic	Core5	0.328	0.0208	0.688	0.142
Lower RT	Core4	0.227	0.0219	0.701	0.142
Upper RT	Core3	-0.332	0.0236	0.731	0.142
Lower RT	Core3	-0.245	0.0345	1	0.178
Lower RT	Core6	0.243	0.0489	1	0.22

Table S13: PICFLU absolute serum cores and individual cytokine associations with phenotypes

Outcome	Core	Coef	P-value	FWER	FDR
Shock	Core5	2.62	5.26e-07	**1.11e-05**	1.11e-05
Shock	Core6	2.31	2.33e-05	**0.000466**	0.000245
ECMO or Death	Core6	2.18	0.00104	**0.0197**	0.00727
Pneumonia-ARDS	Core5	1.52	0.0105	0.189	0.0467
Pneumonia-ARDS	Core6	1.57	0.0111	0.189	0.0467
ECMO or Death	Core5	1.71	0.0184	0.294	0.0643
ECMO or Death	Core2	1.6	0.0256	0.383	0.0749
ECMO or Death	Core1	0.643	0.0286	0.4	0.0749

Table S14: PICFLU adjusted serum cores and individual cytokine associations with phenotypes

Outcome	Core	Coef	P-value	FWER	FDR
Shock	Core5	2.57	1.08e-06	**1.94e-05**	1.94e-05
Pneumonia-ARDS	Core5	1.99	8.95e-05	**0.00152**	0.000805
ECMO or Death	Core1	0.402	0.00022	**0.00352**	0.00132
Shock	Core1	0.534	0.000381	**0.00572**	0.00167
Shock	Core6	1.82	0.000465	**0.00651**	0.00167
ECMO or Death	Core5	2.65	0.000601	**0.00781**	0.0018
Pneumonia-ARDS	Core1	0.653	0.00891	0.107	0.0229
Pneumonia-ARDS	Core2	0.726	0.04	0.44	0.09

Table S15: SHIVERS adjusted serum cores and individual cytokine associations with phenotypes

Outcome	Core	Coef	P-value	FWER	FDR
SARI	Core5	2.95	0.0465	0.279	0.279

REFERENCES

Oshansky, C. M., Gartland, A. J., Wong, S.-S., Jeevan, T., Wang, D., Roddam, P. L., et al. (2014). Mucosal immune responses predict clinical outcomes during influenza infection independently of age and viral load. *American journal of respiratory and critical care medicine* 189, 449–462. doi:10.1164/rccm.201309-1616OC
